# Fast confocal fluorescence imaging in freely behaving mice

**DOI:** 10.1038/s41598-018-34472-x

**Published:** 2018-11-02

**Authors:** Clara Dussaux, Vivien Szabo, Yan Chastagnier, Jozsua Fodor, Jean-François Léger, Laurent Bourdieu, Julie Perroy, Cathie Ventalon

**Affiliations:** 1 Institut de biologie de l’École Normale Supérieure (IBENS), École Normale Supérieure, CNRS, INSERM, PSL Research University, 46 rue d’Ulm, Paris, 75005 France; 2 IGF, Univ. Montpellier, CNRS, INSERM, 141 rue de la Cardonille, Montpellier, 34094 France

## Abstract

Fluorescence imaging in the brain of freely behaving mice is challenging due to severe miniaturization constraints. In particular, the ability to image a large field of view at high temporal resolution and with efficient out-of-focus background rejection still raises technical difficulties. Here, we present a novel fiberscope system that provides fast (up to 200 Hz) background-free fluorescence imaging in freely behaving mice over a field of view of diameter 230 *μ*m. The fiberscope is composed of a custom-made multipoint-scanning confocal microscope coupled to the animal with an image guide and a micro-objective. By simultaneously registering a multipoint-scanning confocal image and a conventional widefield image, we subtracted the residual out-of-focus background and provided a background-free confocal image. Illumination and detection pinholes were created using a digital micromirror device, providing high adaptability to the sample structure and imaging conditions. Using this novel imaging tool, we demonstrated fast fluorescence imaging of microvasculature up to 120 *μ*m deep in the mouse cortex, with an out-of-focus background reduced by two orders of magnitude compared with widefield microscopy. Taking advantage of the high acquisition rate (200 Hz), we measured red blood cell velocity in the cortical microvasculature and showed an increase in awake, unrestrained mice compared with anaesthetized animals.

## Introduction

Since its early use in the 1960’s, fluorescence microscopy and its application to neuroscience have dramatically developed^1,2^. Moved by an increasing variety of optogenetic tools allowing real time monitoring and perturbation of neuronal activity in living animals^3,4^, key microscope features have been continuously refined. However, suitability for investigation in awake and behaving rodents has restrained for years a wider use of fluorescence microscopy in neuroscience. In an attempt to relax technical constraints hindering such studies, the accessible behavioural range can be restricted such that regular upright microscopes can be used with head-fixed rodents with only minor modifications^5^. Alternatively, microscopes can be redesigned entirely to become suitable for investigation in freely behaving rodents^6–13^. A considerable interest from the neuroscience community has recently prompted substantial expansion of this later strategy, despite the technical difficulties arising from severe miniaturization constraints. In particular, imaging at high temporal resolution, over a large field of view and with efficient out-of-focus background rejection (optical sectioning) is challenging. A high acquisition rate is necessary for capturing fast events correlating with neuronal activity such as blood flow^14^, intracellular calcium or membrane voltage changes^15^. Recording from large fields of view is determinant to analyse biological functions over statistically significant ensembles or study networks properties. Finally, background fluorescence rejection, as provided by confocal, light sheet or two-photon microscopy, is a key requirement for performing three-dimensional imaging in densely labelled and thick brain regions^1,16^. All of these features are therefore essential to perform functional recordings in neuroscience.

To implement fluorescence imaging in freely behaving rodents, microscopes have been miniaturized to be small and light enough to be carried by the animal^7^:

1. Miniature single-photon widefield microscopes^6^ have been successfully used in several laboratories for functional imaging in various brain areas^17^. While they benefit from extremely large fields of view of about 650 × 900 μm^18^, they have only limited acquisition rates of about 50 Hz, and they lack optical sectioning.
2. Miniature two-photon microscopes provide good background rejection and are therefore well-suited for imaging densely labelled and thick tissues. Besides, they allow imaging at higher depths, up to several hundred micrometers. However, they have limited fields of view (<130 μm) and acquisition rates (<40 Hz)^8,9^. Finally, because miniature single- and two-photon microscopes must remain very light (<2 g for a mouse), complexity of the optical design is limited, which has prevented so far implementation of simultaneous imaging and targeted optical photostimulation.

To address the possibility to image at high frame rates, over large fields of view and with optical sectioning, we propose to rely on an alternative approach based on image guides. Image guides act as a relay between a regular-sized microscope and the rodent brain, such that the head-mounted part merely contains the distal end of the image guide and a miniaturized objective^10–13^. Therefore, miniaturization constraints are alleviated and sophisticated techniques can be implemented. Based on this configuration, we have previously designed a system allowing for simultaneous functional imaging and targeted photoactivation, with which we demonstrated selective photoactivation of individual neurons in freely behaving mice^13^. Fluorescence imaging, however, was performed using structured illumination microscopy (SIM) or scanless multipoint confocal microscopy and suffered either from movement artefacts and large shot noise with SIM, or very limited sampling with scanless confocal microscopy. In the present work, we have developed a novel fiberscope providing background-free imaging at high acquisition rates over large fields of view, with limited shot noise and motion artefacts. The implemented technique is called differential multipoint-scanning confocal imaging. It is based on a regular multipoint-scanning confocal microscope for which residual background originating from cross-talk between multiple illumination pinholes is subtracted thanks to the simultaneous collection of a standard widefield image^19–21^. Our practical implementation makes use of a Digital Micro-mirror Device (DMD) to achieve high adaptability to experimental requirements. Using this setup, we demonstrated fluorescence imaging of neocortical microvasculature over a field of view of 230 μm, at speeds up to 200 Hz and depths up to 120 μm in freely behaving mice. Out-of-focus background (relative to in-focus signal) was reduced by two orders of magnitude compared with widefield microscopy. Taking advantage of these features, we measured red-blood cells velocity in the same microvessels during anaesthesia and unrestrained behaviour and showed an increase of this velocity in freely behaving mice.

## Theory

### Description of the differential multipoint-scanning confocal microscope

Multipoint-scanning confocal imaging consists in illuminating the sample with an array of light points created with a matrix of pinholes, and detecting fluorescence through the same matrix^22^. The matrix is scanned rapidly to illuminate the full field of view during acquisition of a single image with a camera. This system allows for a significant improvement in imaging speed compared to conventional confocal microscopy, but this gain comes at the expense of background rejection^23^. To quantify this effect, we can express the intensity detected at the camera *I*_*d,*__conf_ (in W/m^2^) as a function of the fluorophore concentration in the object (*O*) and the parameters of the optical system. By using the model described in^24^, neglecting the pixelation artefacts from the DMD and employing the formalism and normalization factors from Mertz^25^, we obtain:

1 $$\begin{array}{cll}{I}_{d,{\rm{conf}}}({x}_{d},\,{y}_{d}) & = & {\rm{\Omega }}{\sigma }_{f}{T}_{{\rm{conf}}}{\iiint }_{-\infty }^{+\infty }{{\rm{PSF}}}_{{\rm{d}}}({x}_{d}-x,\,{y}_{d}-y,\,z)\\  &  & \times {I}_{i,{\rm{conf}}}(x-{x}_{d},\,y-{y}_{d},\,z)O(x,\,y,\,z){\rm{d}}x{\rm{d}}y{\rm{d}}z\end{array}$$

where Ω is the solid angle of the objective entrance pupil as seen by the object, *σ*_*f*_ is the fluorescence excitation cross section, *T*_conf_ is the transmission of the detection optics, PSF_d_ is the detection point spread function (PSF) of the microscope objective, and *I*_*i,*__conf_ is the illumination intensity (expressed in W/m^2^).

*I*_*i,*__conf_ depends on the matrix of illumination pinholes *G* (which is identical to the matrix of detection pinholes), the illumination point-spread function of the microscope objective PSF_i_ and the time-averaged illumination density at the sample *I*_*i*_. For an incoherent light source, it can be expressed as:

2 $${I}_{i,{\rm{c}}{\rm{o}}{\rm{n}}{\rm{f}}}(x,\,y,\,z)={I}_{i}{\iint }_{-{\rm{\infty }}}^{+{\rm{\infty }}}G(x^{\prime} ,\,y^{\prime} ){{\rm{P}}{\rm{S}}{\rm{F}}}_{{\rm{i}}}(x-x^{\prime} ,\,y-y^{\prime} ,\,z){\rm{d}}x^{\prime} {\rm{d}}y^{\prime} $$

In the present work, illumination is produced by transmitting a coherent light beam through an image guide with multimode individual fibers, resulting in an illumination intensity distribution with significant spatial inhomogeneities (speckle) (Fig. S1A) and with an envelope following equation 2. Because the spatial inhomogeneities are averaged out by the detection PSF, the theory can be simplified by considering incoherent beams. The main consequence of these inhomogeneities is to increase the acquisition noise (see SI text).

**Figure 1:**
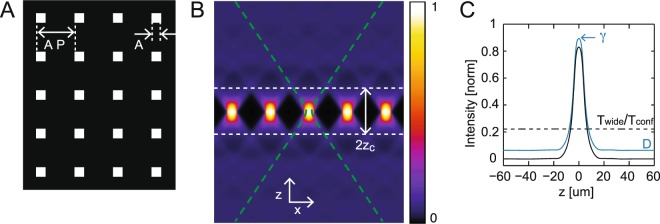
Principle of the optical method and expected optical sectioning. (**A**) The illumination and detection grid *G* is composed of square pinholes of size *A* arranged along a rectangular grid with a distance of *AP* between pinholes (*P* = 4 in this case). (**B**) Illumination intensity for multipoint-scanning confocal imaging *I*_*i*,conf_ plotted along *x* and *z*, corresponding to the illumination grid plotted in A. For |*z*| < *z*_*c*_ (region inside the dotted white lines), the illumination cones corresponding to different pinholes are well-separated. The envelope of the detection PSF of the microscope objective is also shown (dotted green line). (**C**) Expected signals for a fluorescent plane as a function of its position *z*_*s*_ for widefield imaging (dotted black line), regular (solid blue line) and differential (solid black line) multipoint-scanning confocal imaging. Signals were normalized by the constant *I*_0_ (equation 17). We chose a ratio between transmission of the detection optics in the confocal and widefield pathways *T*_wide_/*T*_conf_ = 1/4.5 (value corresponding to our optical setup). For regular multipoint-scanning imaging, the signal measured for |*z*| > 20 μm is equal to the pinhole density *D* (*D* = 1/*P*^2^ ≈ 0.06).

In our experiments, *G* is a regular 2-dimensional array of square pinholes of size *A* separated by a distance *AP* (see Fig. 1A):

3 $$G(x,\,y)=\sum _{i,\,j\in {\mathbb{N}}}{{\rm{rect}}}_{{\rm{A}}}(x-iAP,\,y-jAP)$$

where rect_A_(x, y) = 1 if |*x*| < *A*/2 and |*y*| < *A*/2, and rect_*A*_(*x*, *y*) = 0 otherwise.

For comparison, the detected intensity for a widefield microscope *I*_*d,*__wide_ follows the same expression as equation 1 with *I*_*i,*__wide_(*x*_*d*_, *y*_*d*_, *z*) = *I*_*i*_ (uniform illumination):

4 $${I}_{d,{\rm{w}}{\rm{i}}{\rm{d}}{\rm{e}}}({x}_{d},\,{y}_{d})={\rm{\Omega }}{\sigma }_{f}{T}_{{\rm{w}}{\rm{i}}{\rm{d}}{\rm{e}}}{I}_{i}{\iiint }_{-{\rm{\infty }}}^{+{\rm{\infty }}}{{\rm{P}}{\rm{S}}{\rm{F}}}_{{\rm{d}}}({x}_{d}-x,\,{y}_{d}-y,\,z)O(x,\,y,\,z){\rm{d}}x{\rm{d}}y{\rm{d}}z$$

To further analyse the differences between the two techniques, we can divide the illumination intensity of the multipoint-scanning confocal microscope in two axial regions (Fig. 1B). For |*z*| < *z*_*c*_, illumination is composed of well-separated illumination cones produced by the individual pinholes, with only one of them overlapping with PSF_d_. For |*z*| > *z*_*c*_ the overlap of different illumination cones generates an illumination that can be approximated as uniform - therefore, illumination intensity is similar to that of a widefield microscope, except multiplied by the pinhole density *D* (*D* = 1/*P*^2^) corresponding to the ratio between the surface covered by pinholes and the total surface of the grid. We can thus write *I*_*d*,conf_ as the sum of a signal originating from regions |*z*| < *z*_*c*_ and a background originating from |*z*| > *z*_*c*_:

5 $${I}_{d,{\rm{conf}}}={T}_{{\rm{conf}}}({S}_{{\rm{conf}}}+DB)$$

with

6 $$\begin{array}{lll}{S}_{{\rm{conf}}}({x}_{d},\,{y}_{d}) & = & {\rm{\Omega }}{\sigma }_{f}{I}_{i}{\iiint }_{x,y\in {\mathbb{R}},|z| < {z}_{c}}{{\rm{PSF}}}_{{\rm{d}}}({x}_{d}-x,\,{y}_{d}-y,\,z)\\  &  & {\times {\rm{PSF}}}_{i,{\rm{conf}}}(x-{x}_{d},\,y-{y}_{d},\,z)\,O(x,\,y,\,z){\rm{d}}x{\rm{d}}y{\rm{d}}z\end{array}$$

and

7 $$B({x}_{d},\,{y}_{d})={\rm{\Omega }}{\sigma }_{f}{I}_{i}{\iiint }_{x,y\in {\mathbb{R}},|z| > {z}_{c}}{{\rm{PSF}}}_{{\rm{d}}}({x}_{d}-x,\,{y}_{d}-y,\,z)O(x,\,y,\,z){\rm{d}}x{\rm{d}}y{\rm{d}}z$$

In equation 6, PSF_i,conf_ is the illumination PSF of the multipoint-scanning confocal microscope for regions |*z*| < *z*_*c*_, which is similar to that of a regular confocal microscope with square pinhole illumination:

8 $${{\rm{P}}{\rm{S}}{\rm{F}}}_{i,{\rm{c}}{\rm{o}}{\rm{n}}{\rm{f}}}(x,\,y,\,z)={\iint }_{\begin{array}{c}-{\rm{\infty }}\end{array}}^{+{\rm{\infty }}}{{\rm{r}}{\rm{e}}{\rm{c}}{\rm{t}}}_{{\rm{A}}}(x^{\prime} ,\,y^{\prime} ){{\rm{P}}{\rm{S}}{\rm{F}}}_{{\rm{i}}}(x-x^{\prime} ,\,y-y^{\prime} ,\,z){\rm{d}}x^{\prime} {\rm{d}}y^{\prime} $$

Similarly, we can rewrite the widefield signal as:

9 $${I}_{d,{\rm{wide}}}={T}_{{\rm{wide}}}({S}_{{\rm{wide}}}+B)$$

with

10 $${S}_{{\rm{wide}}}({x}_{d},{y}_{d})={\rm{\Omega }}{\sigma }_{f}{I}_{i}{\iiint }_{x,y\in {\mathbb{R}},|z| < {z}_{c}}{{\rm{PSF}}}_{{\rm{d}}}({x}_{d}-x,{y}_{d}-y,z)O(x,y,z){\rm{d}}x{\rm{d}}y{\rm{d}}z$$

By acquiring the two images simultaneously (with the same illumination intensity *I*_*i*_), it is then possible to subtract the residual background obtained with multipoint-scanning confocal imaging, yielding a background-free image of intensity *I*_*d,*__diff_:

11 $${I}_{d,{\rm{diff}}}={I}_{d,{\rm{conf}}}-D\frac{{T}_{{\rm{conf}}}}{{T}_{{\rm{wide}}}}{I}_{d,{\rm{wide}}}$$

12 $${I}_{d,{\rm{d}}{\rm{i}}{\rm{f}}{\rm{f}}}={T}_{{\rm{c}}{\rm{o}}{\rm{n}}{\rm{f}}}({S}_{{\rm{c}}{\rm{o}}{\rm{n}}{\rm{f}}}-D{S}_{{\rm{w}}{\rm{i}}{\rm{d}}{\rm{e}}})$$

We refer to this technique as differential multipoint-scanning confocal imaging.

### Considerations on signal and noise

From equations 11 and 12, we can draw 2 important remarks:

1. By subtracting a fraction of the widefield image to the multipoint-scanning confocal image, we eliminate the background but we also decrease the in-focus signal (Eq. 12). The value of *D* can be chosen such that this decrease in signal remains small.
2. We can quantify the standard deviation of the noise σ_*d*,diff_ obtained with differential multipoint-scanning confocal imaging by considering that the number of detected photons follows a Poisson distribution, and that the associated shot noise is the main source of noise. The number of photons detected on a camera pixel of size *A*_*p*_ during the exposure time *t* can be expressed as *αI*_*d*_ where $\alpha ={A}_{p}^{2}t/(h\nu )$ (with *v* the photon frequency). We then deduce:

13 $${{\sigma }_{d,{\rm{diff}}}}^{2}=\alpha {I}_{d,{\rm{conf}}}+{(D\frac{{T}_{{\rm{conf}}}}{{T}_{{\rm{wide}}}})}^{2}\alpha {I}_{d,{\rm{wide}}}$$

ie.

14 $${{\sigma }_{d,{\rm{diff}}}}^{2}={T}_{{\rm{conf}}}\alpha ({S}_{{\rm{conf}}}+{D}^{2}\frac{{T}_{{\rm{conf}}}}{{T}_{{\rm{wide}}}}{S}_{{\rm{wide}}})+D{T}_{{\rm{conf}}}\alpha (1+D\frac{{T}_{{\rm{conf}}}}{{T}_{{\rm{wide}}}})B$$

In this latter equation, the first term in each parenthesis originates from the confocal image and the second term from the widefield image. The parameters of the optical setup (*T*_conf_/*T*_wide_ and *D*) can be chosen such that the contribution from the widefield image to the noise remains small compared to the contribution from the confocal image. The subtraction process then leaves the noise mostly unchanged. This consideration was a major criteria when designing our optical setup.

### Predictions for a fluorescent plane

To characterize the optical sectioning of the different techniques, intensity from a fluorescent plane can be measured as a function of its axial position *z*_*s*_ (*O*_*plane*_ = *O*_0_*δ*(*z* − *z*_*s*_)). Using equations 1 and 4, we can plot the expected intensity in the case of regular multipoint-scanning confocal imaging and widefield imaging (Fig. 1C). For multipoint-scanning confocal imaging the maximum intensity is obtained in *z*_*S*_ = 0 and is equal to:

15 $${I}_{d,\mathrm{conf},{\rm{plane}}}({z}_{s}=0)=\gamma {\rm{\Omega }}{\sigma }_{f}{I}_{i}{T}_{{\rm{conf}}}{O}_{0}=\gamma {I}_{0}$$

with

16 $$\gamma ={\iint }_{-\infty }^{+\infty }{{\rm{PSF}}}_{{\rm{d}}}(x,\,y,\,\mathrm{0)}{{\rm{PSF}}}_{i,{\rm{conf}}}(x,\,y,\,\mathrm{0)}{\rm{d}}x{\rm{d}}y$$

and

17 $${I}_{0}={\rm{\Omega }}{\sigma }_{f}{I}_{i}{T}_{{\rm{conf}}}{O}_{0}$$

*γ* is the overlap integral between PSF_i,conf_ and PSF_d_. Its maximum value is equal to 1 and is obtained when the pinhole size is significantly larger than the size of PSF_i_ and PSF_d_. It decays in the presence of aberrations.

As expected, for large values of *z*_*s*_, fluorescence intensity drops to a constant baseline:

18 $${I}_{d,\mathrm{conf},{\rm{plane}}}(|{z}_{s}| > {z}_{c})=D{I}_{0}$$

For comparison, the intensity detected with widefield microscopy does not depend on *z*_*s*_:

19 $${I}_{d,\mathrm{wide},{\rm{plane}}}({z}_{s})=({T}_{{\rm{wide}}}/{T}_{{\rm{conf}}}){I}_{0}$$

In practice, measurement of these optical sectioning curves allows calibrating the fraction of the widefield image that needs to be subtracted to obtain a background-free differential multipoint-scanning confocal image (equation 11):

20 $$D\frac{{T}_{{\rm{conf}}}}{{T}_{{\rm{wide}}}}=\frac{{I}_{d,\mathrm{wide},{\rm{plane}}}({z}_{s})}{{I}_{d,\mathrm{conf},{\rm{plane}}}(|{z}_{s}| > {z}_{c})}$$

Finally, we computed the expected signal from a fluorescent plane for differential multipoint-scanning confocal imaging (Fig. 1C). We obtained a background-free signal with a maximum value at *z* = 0 equal to:

21 $${I}_{d,\mathrm{diff},{\rm{plane}}}({z}_{s}=0)=(\gamma -D){I}_{0}$$

To summarize this theory section, differential multipoint-scanning confocal imaging is a single-shot technique for which out-of-focus background is completely removed, thus producing high-contrast images. This gain in contrast implies some degradation of the signal and noise compared to a regular multipoint-scanning confocal image, but the parameters of the setup can be chosen such that this degradation remains very small.

## Results

### Optical implementation and characterization of the fiberscope

#### Implementation of the technique

In a classical implementation of multipoint-scanning confocal imaging, the pinhole matrix *G* is imprinted on a disk that is rotated at high speed (*spinning disk microscopy*). In our case, as in^20,26^, this matrix is created by intensity modulation using a DMD. The fast refreshing rate of DMDs (16.4 kHz with the Vialux V-9601 VIS module) allows displacing the pinhole matrix at high speed, with no moving part. The key advantage of this implementation is that the size and density of pinholes can be easily adapted to the sample under investigation.

**Figure 2:**
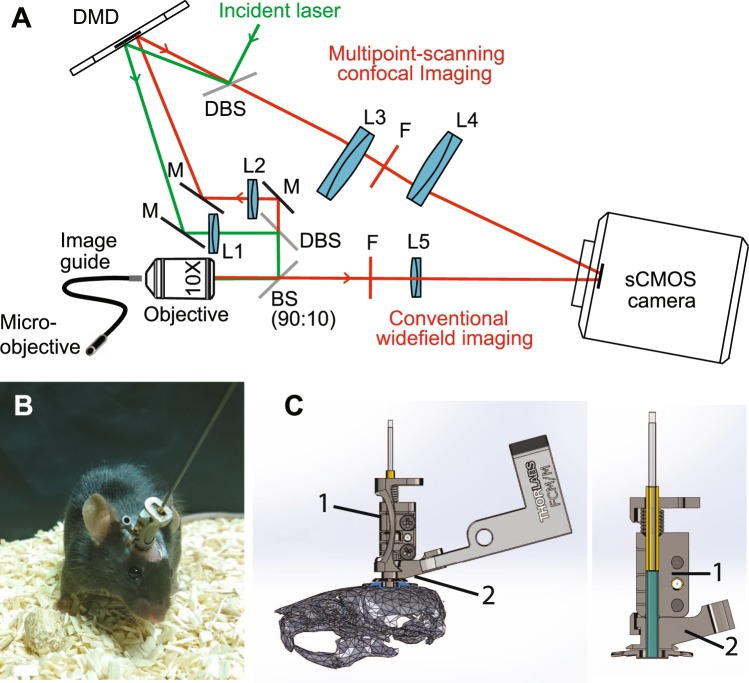
Optical setup. (**A**) A custom-made microscope comprising one illumination path (green) and two detection paths (red) is coupled to the animal brain using an image guide attached to a micro-objective. The central element of the fiberscope setup is a DMD, which is used to create a matrix of illumination and detection pinholes. The optical setup is fully described in the methods. Lenses L_3_ and L_4_ are off-axis to partly straighten up the multipoint-scanning confocal image at the camera. DBS: dichroic beam splitter. M: mirror. BS(90:10): beam splitter with 90% reflection and 10% transmission. F: filter. (**B**) Picture of an unrestrained (freely behaving) mouse in its cage with the fiberscope probe fixed on the skull. (**C**) Fixation of the image guide (ferrule shown in yellow) and GRIN lens (ferrule shown in cyan) to the skull. The image guide is attached to the GRIN lens using a connection device (piece 1). The GRIN lens is fixed to the skull using a head plate (piece 2) that is positioned using a micromanipulator.

The optical setup is shown on Fig. 2A and fully described in the methods. Briefly, a 561nm laser beam is reflected on the left part of a DMD displaying a sequence of illumination pinhole matrices similar to the pattern shown on Fig. 1A. These intensity patterns are then imaged onto the entrance surface of an image guide (Fujikura FIGH-30-650S), transported in the image guide and reimaged at a distal plane in the mouse brain using a micro-objective (Grintech 01 NEM-100-25-10-860-DS-ST). Fluorescence collected by the micro-objective is transported in the image guide to our custom-made microscope where it is separated into two parts. 90% of the beam is imaged on the right part of the DMD (displaying a sequence of detection pinhole matrices), reflected by the DMD, and then reimaged at the camera, producing a multipoint-scanning confocal image. The remaining 10% of the fluorescence beam is directly imaged at the sCMOS camera; because there are no time-varying filter on this second detection path, and because the sum of illumination patterns corresponds to uniform illumination, a conventional widefield image is acquired on this path. The two images are acquired simultaneously on two different regions of the camera.

Separating the laser and the fluorescence at the DMD is useful to optimize the light budget, as described in the methods. It also allows choosing the size of illumination and detection pinholes independently, which is investigated in SI text and Fig. S3. In the main text, illumination and detection pinholes have identical sizes.

Depending on the pinhole density, the maximum imaging rate can be limited either by the camera or the DMD: for *D* > 0.04, it is set by the camera to 330 Hz whereas for *D* < 0.04 it is set by the DMD to *f*_DMD_*D*/2 = *D* * 8.2k Hz (see SI text).

#### Experimental validation of the technique

**Figure 3:**
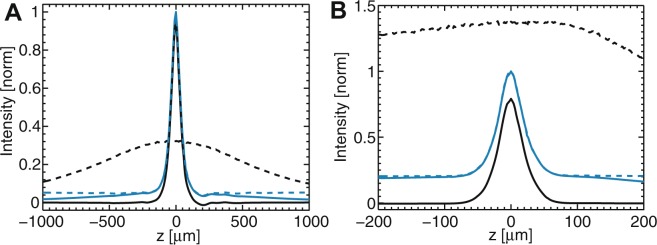
Experimental characterization of the optical sectioning without (**A**) and with (**B**) the fiberscope probe (image guide and micro-objective). Fluorescence intensity from a rhodamine layer is plotted as a function of axial position *z* (*z* = 0 at the objective focal plane) with multipoint-scanning confocal imaging (solid blue line) and conventional widefield imaging (dotted black line). For practical reasons, we used a different normalization compared with the theoretical curves presented in Fig. 1C: here, the intensity measured with widefield and multipoint-scanning confocal imaging were both normalized to the in-focus multipoint-scanning confocal signal. Therefore, the plotted signals are divided by a factor *γ* compared with those plotted in Fig. 1C. The ratio between the two signals (dotted blue line, normalized to 1) yields constant background *D*/*γ* equal to 5% without the fiberscope probe (**A**) and 20% with the probe (**B**), which corresponds respectively to *γ* = 0.8 and *γ* = 0.2 (*D* = 0.04). In both cases, the differential multipoint-scanning confocal signal (solid black line) shows a background equal to zero. Experiments were conducted with identical pinhole patterns at the DMD and therefore different pinhole sizes at the sample: *A* = 19 μm (**A**) and *A* = 7.5 μm (**B**). Pinhole density: *D* = 0.04.

We first performed experiments with a fluorescent plane and compared measurements obtained without (Fig. 3A) and with (Fig. 3B) the fiberscope probe (composed of the image guide and micro-objective). For widefield and regular multipoint-scanning confocal imaging, the shape of the measured optical sectioning curves were similar to that expected from theory, except for a small decay of both signals observed at large values of defocus (instead of constant signals). This decay can be explained by a slight increase of the illumination beam size for large values of defocus, corresponding to a decrease of the excitation density in the central part of the beam. This decay is identical for both techniques, as can be verified by computing the ratio between multipoint-scanning confocal signal and the widefield signal as a function of *z* (dotted blue line, normalized to 1 for *z* = 0). The obtained curve is similar to the theory of multipoint-scanning microscopy, with a constant background for large defocus equal to *D*/*γ*. Therefore, this procedure allows calibrating the value of *γ*, which depends on the parameters of the optical setup. Without the fiberscope probe, we found a value of *γ* close to 1 (*γ*  = 0.8 for the parameters of Fig. 3A) as expected for a pinhole size larger than the PSF size. However, due to severe aberrations from the micro-objective and cross-talk between individual fibres of the image guide, *γ* is significantly smaller when using the fiberscope probe (*γ* = 0.2 with the parameters of Fig. 3B).

We then computed the experimental value of *DT*_conf_/*T*_wide_ using equation 20. We found values of 0.15 and 0.16 with and without the fiberscope probe (respectively), which can be confronted with theoretical value:

22 $$D\frac{{T}_{{\rm{conf}}}}{{T}_{{\rm{wide}}}}=D\frac{1-{T}_{{\rm{BS}}}}{{T}_{{\rm{BS}}}}{R}_{{\rm{DMD}}}$$

where *T*_BS_ is the transmission of the beamsplitter used to separate light into the two detection paths, and *R*_DMD_ is the reflection efficiency of the DMD, which depends on the wavelength and incident angle of the beam. With the parameters from the experimental setup (*T*_BS_ = 0.09, *R*_DMD _= 0.5, *D* = 0.04), we obtain *DT*_conf_/*T*_wide _= 0.20. The slight difference with the experimental value obtained above can be explained by small additional losses (on the order of 20%) on the confocal detection path.

Using the experimental value of *DT*_conf_/*T*_wide_, we finally computed the differential multipoint-scanning confocal signals (solid black lines in Fig. 3A,B). As expected, we obtained background-free signals in both experimental situations (with and without the fiberscope probe), which validates the principle of the technique.

#### Optical characterization of differential multipoint-scanning confocal imaging as a function of pinhole size and density

One advantage of our implementation is that the pinhole size (*A*) and density (*D*) can be easily modified and adapted to the structure of the sample. We characterized three important features of differential multipoint-scanning confocal imaging for a large set of values of *A* and *D*:

1. We measured the illumination power at the sample, for a constant laser power at the DMD. As expected, this power is independent of the pinhole size (data not shown) but varies linearly with the pinhole density (Fig. 4A).

**Figure 4:**
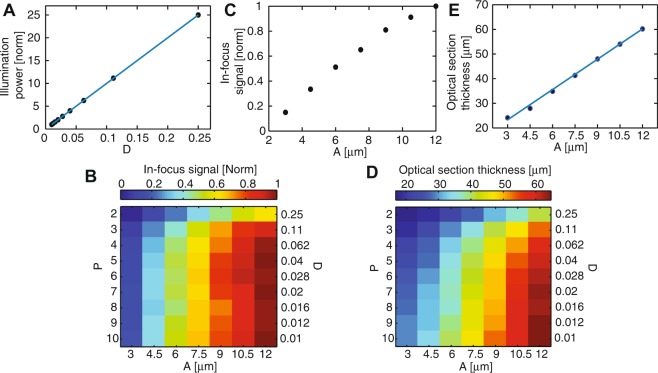
Optical characterization of the differential multipoint-scanning confocal modality. (**A**) Measured illumination power at the sample (dots) as a function of pinhole density *D*, normalized to the value measured for a density of 0.01. As expected from theory, illumination power is linear with *D* (solid blue line). (**B**) Signal measured from an in-focus fluorescent plane as a function of the pinhole size *A* and density *D*, for a constant illumination power of the sample. The signal is normalized to the maximum value, obtained for *A* = 12 μm and *D* = 0.04. (**C**) Linear plot of data in **B**) as a function of *A*, for *D* = 0.04. (**D**) Thickness of the optical section as a function of *A* and *D*. This thickness is calculated as the FWHM of sectioning curves similar to the solid black line in Fig. 3B. (**E**) Linear plot of data in **D**) as a function of *A*, for *D* = 0.04. In the range of pinhole sizes used in this work, section thickness is linear with the pinhole size (solid blue line).
2. We measured the signal obtained with the fluorescent plane in focus. Data was plotted for a constant illumination power at the sample, *i*.*e*. for an illumination intensity at the DMD inversely proportional to *D* (Fig. 4B,C). The obtained in-focus signal is roughly independent of the pinhole density, except for high pinhole densities for which the subtraction procedure leads to a significant decrease of the signal (equation 21). By contrast, it strongly increases with the pinhole size, due to decreased sensitivity to aberrations.
3. We plotted the thickness of the optical sections (Fig. 4D,E), defined as the full-width at half maximum (FWHM) of the optical sectioning curves (solid black lines, Fig. 3B). This thickness varied between 17 and 64 μm depending on the parameters and was approximately linear with the pinhole size (Fig. 4E), as expected from theory^27^. It also slightly decreases when the pinhole density increases, which can be interpreted as follows. For small pinhole densities, it approaches that of single-point-scanning confocal microscopy, as cross-talk between pinholes is limited. By contrast, for large pinhole densities, it is on the order of the axial range over which illumination cones corresponding to different pinholes are well-separated (*z*_*c*_, Fig. 1B). This distance can be made much shorter than the regular confocal section thickness by using small pinhole spacing. However, this gain in section thickness is obtained at the expense of signal (see Fig. 4B and equations 12 and 21).

Using these considerations, we can draw the main influences of the pinhole size and density on the performances of differential multipoint-scanning confocal imaging. Regarding the pinhole size, there is a compromise between in-focus signal (large in-focus signal is obtained for large pinholes) and thickness of the optical section (thin optical section is obtained for small pinholes), similar to what is obtained with regular confocal microscopes. Because with our current optical setup the signal is limited by strong aberrations from the GRIN lenses and cross-talk in the image guide, we use larger pinhole sizes compared with standard implementation of confocal microscopy and thus obtain thicker optical sections. As for the pinhole density, it influences both the maximum imaging rate and the signal to noise ratio. For *D* > *T*_wide_/*T*_conf_, noise coming from the widefield image is larger than noise from the confocal image (equation 13). Conversely, a small density will lead to a small illumination power at the sample and therefore a small collected signal. Therefore, there is an optimal density that maximizes the signal to noise ratio, which can be derived using equation 14 and depends on the optical setup parameters (including aberrations of the micro-objective), sample brightness and amount of background (which in turns depends on fluorescence labelling). An example of this calculation will be given in the next section in the case of microvasculature imaging.

### Application to microvasculature imaging and measurement of red blood cell velocity

#### Background-free imaging of microvasculature

**Figure 5:**
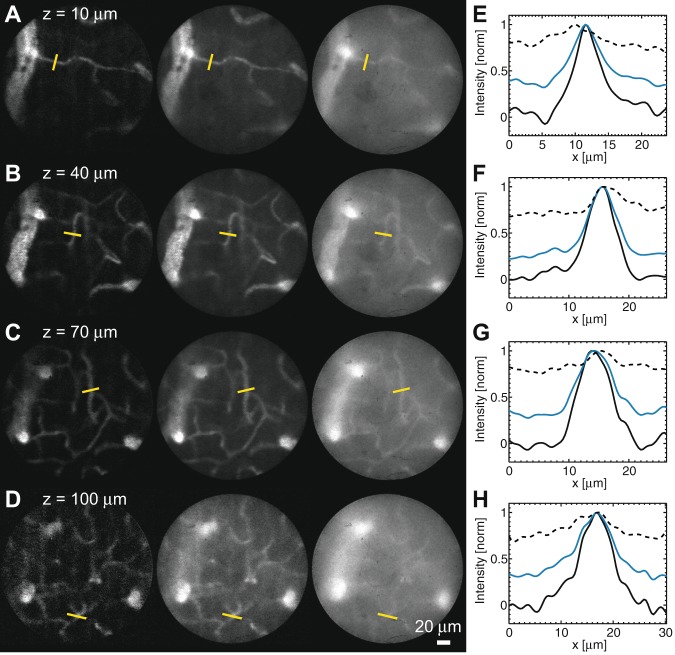
Comparison of different techniques for imaging microvasculature in the cortex of anaesthetized mice following retro-orbital sinus injection of rhodamine dextran. (**A**–**D**) Differential (left) and regular (middle) multipoint-scanning confocal imaging are compared with conventional widefield imaging (right). Images are registered at depths of 10 μm (**A**), 40 μm (**B**), 70 μm (**C**) and 100 μm (**D**) in the brain. (**E–H**) Profiles along the yellow lines drawn in Figs (**A**–**D)** (respectively) measured with differential (solid black line) and regular (solid blue line) multipoint-scanning confocal imaging, and with conventional widefield imaging (dotted black line). Imaging speed: 100 Hz. *A* = 7.5 μm, *D* = 0.04.

We then turned to *in vivo* fluorescence imaging of microvasculature in the mouse cortex (Fig. 5). We first quantified the ratio between the out-of-focus background and the in-focus signal (*B*/*S*) by plotting line profiles across small vessels (diameter <12 μm) at depths up to 100 μm below the brain surface (Fig. 5E–H; see methods for the determination of *S* and *B*). Images acquired with conventional widefield microscopy exhibited a small contrast with an out-of-focus background significantly larger than the signal ((*B*/*S*)_wide_ = 4.6, 95% confidence interval of the mean (CI95) [4.1, 5.2], *n* = 107 vessels from 6 mice). Regular multipoint-scanning imaging of the same microvessels allowed for a significant background decrease ((*B*/*S*)_conf_ = 0.66, CI95 [0.57, 0.74], *n* = 107 vessels from 6 mice). Finally, differential multipoint-scanning confocal imaging showed the highest contrast and background rejection, such that small vessels could be easily visible up to a depth of about 100 μm ((*B*/*S*)_diff_ = 6.7 × 10^−2^, CI95 [4.6 × 10^−2^, 8.9 × 10^−2^], *n* = 107 vessels from 6 mice). In fact, the background to signal ratio was about two orders of magnitude lower with differential multipoint-scanning confocal imaging than with conventional widefield imaging ((*B*/*S*)_diff_/(*B*/*S*)_wide_ = 1.3 × 10^−2^, CI95 [8.7 × 10^−3^, 1.8 × 10^−2^], *n* = 107 vessels from 6 mice). Notably, similar background rejection was observed independently of depth up to 100 μm (Fig. 5).

#### Influence of the pinhole density

As we discussed in the characterization section, the pinhole density strongly influences the signal to noise ratio for differential multipoint-scanning confocal imaging. To further investigate this effect, we calculated this signal to noise ratio as a function of *D* in the case of microvasculature imaging, using the parameters of the optical setup (*T*_conf_, *T*_wide_), and a pixel size of 7.5 μm (SI text). We showed that it is maximum for *D* = 0.04 (Fig. S4). We also found that the loss of signal and the increase of noise compared with regular multipoint-scanning confocal imaging were then limited to respectively 20% and 5% (SI text). Besides, the density could be easily adapted if a different sample was used, or if the characteristics of the optical setup were modified.

#### Influence of the pinhole size

**Figure 6:**
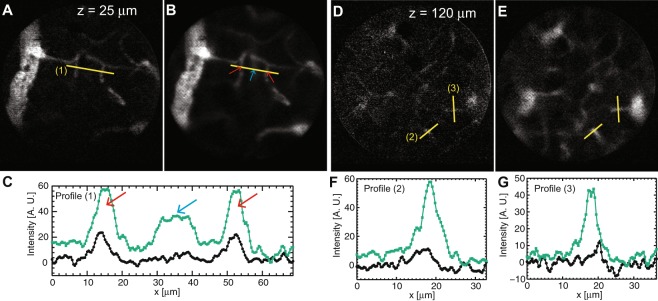
Effect of the pinhole size for imaging microvasculature in anaesthetized mice with differential multipoint-scanning confocal microscopy. (**A**–**C**) Imaging close to the brain surface (*z* = 25 μm) with pinhole sizes of 4.5 μm (**A**) and 10.5 μm (**B**). (**C**) Profile along line (1) (shown on Figs **A**,**B**) measured with *A* = 4.5 μm (green line) and *A* = 10.5 μm (black line). The background at the out-of-focus vessel indicated by the blue arrow divided by the signal at the in-focus vessels indicated by the red arrows was 2x larger with *A* = 10.5 μm than with *A* = 4.5 μm. (**D**–**G**) Same as A-C for imaging far from the brain surface (*z* = 120 μm). (**F**) represents the profiles along line (2) and (**G**) along line (3). Imaging speed: 100 Hz. *D* = 0.04.

We then characterized the influence of the pinhole size on image signal and section thickness for differential multipoint-scanning confocal imaging of microvasculature by comparing two different pinhole sizes (4.5 μm and 10.5 μm) (Fig. 6). We expected a signal approximately 2.7x smaller and a section thickness 1.9x thinner with the smallest pinholes (Fig. 4). Indeed, close to the cortex surface (*z* = 25 μm, Fig. 6A–C) signals measured with the smallest pinholes were approximately 2.1x smaller and background rejection was more efficient, as out-of-focus vessels appeared dimmer. We then increased the imaging depth and we could demonstrate imaging at up to *z* = 120 μm below the brain surface (Fig. 6D–G). At these high depths, signals measured with the smallest pinholes where 3.6x smaller than with the largest pinholes. This higher value can be explained by considering that signals measured with small pinholes decay more rapidly with imaging depth due to a higher sensitivity to optical scattering. Therefore, these results suggest that smaller pinholes are more suitable for imaging near the brain surface (providing thinner optical sections) whereas larger pinholes are more suitable for imaging at large depths (providing an increased robustness to scattering).

#### Stable imaging in freely behaving mice

**Figure 7:**
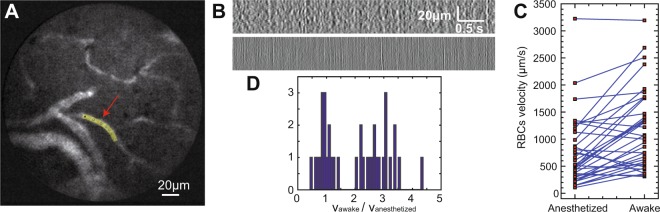
Measurement of RBC velocity in anaesthetized and awake mice. (**A**) Differential multipoint-scanning confocal image acquired in a freely behaving mouse. Exposure time: 5ms. *A* = 6 μm. *D* = 0.04. Time-lapse series is available as Supplemental Movie S1. (**B**) x-t profiles along the region of interest (ROI) indicated by the red arrow and shown in yellow on (**A**) Acquisition rates: 50 Hz (top) and 200 Hz (bottom). Dark streaks on the bottom profiles correspond to RBCs moving in the longitudinal direction, with a velocity directly equal to the slope of the streaks. These streaks could not be observed on the top profiles, showing that an acquisition rate of 50 Hz was too low for reliable extraction of the RBC velocity in this microvessel. (**C**) Comparison of RBC velocity in anaesthetized and in awake, freely behaving mice for 37 microvessels in 5 mice. (**D**) Histogram of the ratio of RBC velocities measured in awake *versus* anaesthetized mice.

We then turned to imaging in freely behaving mice (Fig. 7). In such conditions, one major concern is movements of the field of view during mouse behaviour. By using the movement correction algorithm MOCO^28^, we showed that even though small movements did exist (maximum displacement of the field of view in *x* and *y* was <5 μm, n = 5 mice, Fig. S5), they could easily be corrected, in agreement with what we found in our previous work^13^.

#### Measurement of red blood cells velocity in freely behaving mice

Because fluorescence imaging of microvasculature was performed at high speed and with very limited movement artefacts, this opened the possibility to measure RBC velocity in freely behaving mice. To do so, we plotted x-t profiles along individual microvessels (Fig. 7A,B) and compared the results when imaging at 2 different rates: 50 and 200 Hz. RBC velocity could be easily extracted from the 200 Hz profiles using a Radon-transform algorithm^29^, but not from the 50 Hz profiles. In fact, the maximum RBC speed *v*_max_ that can be measured depends on the length *l* of the vessel portion used to compute the x-t profile and the acquisition rate *f*: *v*_max_ = *lf*/2 (we considered that each RBC should be visible on at least two consecutive frames). For a length of 40 μm and an acquisition rate of 200 Hz, the maximum velocity that can be measured is 4 mm/s, which is larger than RBC velocities in microvasculature^30^.

Using this method, we measured RBC velocity in 37 vessels from 5 different mice (Fig. 7C). In animals anaesthetized with isoflurane (1.5–2.5%), we found a mean velocity of 0.74 mm/s (s.d. 0.63 mm/s). After awakening, mean velocity measured in the same microvessels was 1.1 mm/s (s.d. 0.74 mm/s), showing a mean increase *v*_awake_/*v*_anesthetized_ = 2.1 (s.d. 1.1, Fig. 7D).

## Discussion

We developed a novel fiberscope system that allows high-speed background-free fluorescence imaging in the brain of freely behaving mice. Out-of-focus background relative to in-focus signal was 2 orders of magnitude smaller than with conventional widefield microscopy, enabling high-contrast imaging of blood vessels at speeds up to 200 Hz and depths up to 120 μm in the cortex of unrestrained mice. Moreover, the technique was implemented using a DMD, providing high adaptability to the sample characteristics (staining, scattering) or to the imaging depth. We showed that background rejection is of paramount importance for detecting in-focus objects such as microvessels within thick and densely labelled tissue. It would also be highly beneficial for calcium or voltage imaging as it would allow removing contamination from time-varying out-of-focus events.

Background rejection was obtained in two steps. The first step is based on a regular multipoint-scanning confocal approach, and leads to an improvement in the background to signal ratio by one order of magnitude. Residual background due to cross talk between multiple pinholes is then subtracted in a second step, using simultaneous acquisition of a conventional widefield image. The resulting gain in the background to signal ratio is also of one order of magnitude. The first step comes with a decrease of the signal because of reflection losses at the DMD, cross-talk within the image guide, and aberrations from the GRIN-lens based micro-objective. However, the use of novel micro-objectives of larger NA (NA = 0.8) and smaller aberrations similar to the objective developed by Matz *et al*.^31^ will lead to strong improvements in the detected signal. The second background rejection step could also lead to a decrease in the signal but in our experiments this loss was limited to 20%. Besides, another potential concern with numerical background subtraction is increased of the imaging noise, but we carefully chose the parameters of the optical setup such that this increase was limited to 5%.

Because imaging could be performed at high speeds, we were able to measure RBC velocity in the mouse cortex, finding mean values consistent with the literature^12,32^. Besides, we were able to follow blood flow in the same vessels during anaesthesia and unrestrained behaviour, showing a mean increase in RBC speed of a factor of 2 during waking. Overall, these results show that our system allows for fast events recordings during various experimental conditions, suitable for physiological recordings during natural behaviours or in pathological situations.

Other techniques have been implemented for fluorescence imaging in freely behaving mice, but they present important limitations compared to the technique demonstrated here. Both conventional widefield fiberscopes^10–12^ and miniature microscopes^6^ lack optical sectioning. Besides, current implementations of miniature microscopes are limited to 50 Hz acquisition rates. Miniature two-photon microscopes provide high resolution and background rejection but are limited to a small field of view (130 μm) and speed (40 Hz). Finally, we have previously developed a fiberscope allowing structured-illumination and scanless confocal fluorescence imaging^13^. However, structured-illumination imaging is prone to motion artefacts and provides limited signal to noise ratios, and scanless confocal imaging is limited to a few points in the field of view.

Our method can also be compared with other fiberscope systems that have been developed for biological tissue imaging with high background rejection but that have never been applied to imaging in freely behaving mice^33–40^. The implemented techniques suffer from a few drawbacks compared to the method developed in this paper: Single point-scanning confocal^33^ and two-photon^34^ imaging are limited in speed; HiLo^35^ and differential structured illumination^36^ microscopy suffer from the same limitations in signal to noise ratio than structured illumination microscopy^13,41^; Line-^37–39^ and multipoint-^40^ scanning confocal imaging give access to high-speed imaging with high signal to noise ratios, but background rejection is less efficient than with our differential multipoint-scanning confocal fiberscope. Finally, differential line-scanning confocal imaging^38^ provides background-free imaging with high signal to noise ratios, but this technique is not single shot and therefore is slower than the technique demonstrated in this paper (maximum frame rate is 60 Hz) and prone to motion artefacts.

A question of particular interest is the applicability of our setup to image neuronal activity, using either calcium indicators or voltage sensors. For conducting such experiments, a strong requirement is a high signal to noise ratio (SNR). Optical sectioning provided by our setup reduces significantly the noise, as background photons (and the associated noise) are not recorded, as compared to simpler techniques such as conventional widefield miniature fluorescence microscopes. However, imperfect reflection at the DMD, aberrations of the GRIN lenses and cross talk in the image guide cause a significant signal loss. In addition, the use of coherent illumination beams generates speckle noise, which can nevertheless be limited by rapidly modifying the speckle pattern (SI text and Fig S1). Therefore, applicability of our fiberscope to functional imaging will depend on achievable imaging speed and SNR *in vivo*, two antagonist parameters for which a compromise will need to be found. In the future, novel micro-objectives with high NA and low aberrations will allow collecting more photons and improving the SNR^31^. Another requirement for imaging neuronal activity is a spatial resolution compatible with cellular resolution. With our fiberscope, the resolution is 2 μm laterally and 10.5 μm axially (Fig. S2), which should be sufficient to meet this criteria in many brain regions. In regions where cellular density is high, sparse labeling could be used^42^, and post-processing algorithms could help disambiguate signals from neighbor cells^43,44^. Along the same line, signals coming from somata can be mixed with signal coming from the neuropil, a problem that is encountered with most optical imaging tools, including 2-photon imaging^42,45^. Efficient algorithms have been developed to remove this neuropil contamination and could be readily applied to data acquired with our fiberscope^43–45^. Lastly, because the maximum imaging depth demonstrated with our setup is about 120 μm, imaging could be performed in the neocortex down to the top of layer 2, in the superficial layers of the olfactory bulb, and the molecular layer of the cerebellum, while keeping the observed brain region intact. Besides, at cost of grin lens insertion, imaging could be performed in the pyramidal layer of CA1, and, using smaller grin lenses than the one used here, in deeper brain regions such as the thalamus or hypothalamus. Use of smaller grin lenses does not require any modification of the optical setup and should not cause any degradation in optical performances except for a small reduction of the field of view (210 μm for 500 μm diameter lenses).

Finally, one major advantage of our fiberscope is that it is compatible with targeted photoactivation of channelrhodopsin 2 (ChR2), either using computer generated holography^13^ or using fast intensity modulation with a DMD. Such a system would then open the way to studies of neurovascular coupling using highly precise spatiotemporal sequences of neuronal activity, and enable all-optical electrophysiology in freely behaving mice by simultaneous full-field calcium imaging of red GECI and targeted photoactivation of ChR2.

## Methods

### Optical setup

#### Design and optimization

The DMD can be modelled as a 2 dimensional blazed grating, with a diffraction efficiency into the main order strongly dependent on the incident angle and the wavelength^46^. Therefore, we chose to separate the laser and fluorescence beams on the surface of the DMD and optimize their angles independently (see Fig. 2). For the laser beam, the angle between the beam reflected at the DMD and the normal to the DMD is equal to 13.5°. For the fluorescence beam, the angle between the beam incident on the DMD and the normal to the DMD is equal to 9°. These angles allow reaching diffraction efficiency into the main order close to 60% for both beams, which is the maximum value that can be achieved with this DMD. Using this configuration, illumination and detection pinholes are located on two sides of the DMD and are independent from one another. Therefore, the size of detection pinholes can be made slightly larger than that of illumination pinholes to increase collection efficiency. In addition, an incident angle of 13.5° is also suitable for a 488nm laser beam. Therefore a second laser beam collinear with the 561nm laser beam could be used, for example, for photoactivation of Channelrhodopsin 2.

A 561nm laser beam (LMX-561L-500-COL-PP, Oxxius) is expanded using a telescope composed of two achromatic doublets (of focal lengths 25 mm and 200 mm, AC127–025-A-ML and AC254-200-A-ML, Thorlabs) and illuminates the left part of the DMD. The DMD is rotated by 45° around its normal such that the reflected beam (in the main diffraction order) lies in the horizontal plane. The DMD is imaged onto the entrance surface of the image guide using a tube lens (*f*_1_ = 150 mm, DLB-20-150PM, Optosigma), and a 10*X* microscope objective (UPLSAPO 10X2, Olympus). In this way, illumination grid patterns displayed at the DMD are projected onto the entrance surface of the image guide, transported in the image guide and then reimaged in the sample using a micro-objective (01 NEM-100-25-10-860-DS-ST, Grintech). Fluorescence collected by the micro-objective is imaged onto the image guide and transported to the microscope, where it is split into two parts using a 90/10 beamsplitter (21011, 90/10 Beamsplitter - UF2, Chroma) placed after the objective. 90% of the fluorescence beam is imaged (using the objective and a tube lens (*f*_2_ = 150 mm, DLB-20-150PM, Optosigma)) onto the right part of the DMD, displaying a pattern of detection pinholes. Fluorescence light reflected at the DMD is then imaged on the left part of the camera using a relay lens composed of 2 identical relay lenses (*f*_3_ = *f*_4_ = 150 mm, AC508-150-A, Thorlabs), forming a multipoint-scanning confocal image. The remaining 10% of fluorescence light transmitted by the 90/10 beamsplitter is directly imaged onto the right part of the camera (using the microscope objective and a tube lens (*f*_5_ = 125 mm, AC254-125-A-ML, Thorlabs)) forming a conventional widefield image. Confocal and widefield images are acquired simultaneously. Emission filters are placed on the two detection paths to reject residual laser light.

The detection beam path between the DMD and the camera was carefully optimized, by taking into account one important constraint, namely that detection efficiency of the camera drops for incident angles larger than 18° (data not shown). This is due to the structure of sCMOS pixels, that contain a microlens to focus light on a small detector. For large incidence angle, a part of light does not reach the sensitive area. Therefore, we made sure that the incidence angle on the camera for the multipoint-scanning confocal image was smaller than 18°. Since the angle between the DMD surface and the fluorescence beam reflected at the DMD is larger than this value (33°), we used the off-axis relay lens formed by lenses *L*_3_ and *L*_4_ to partly straighten up the image (anamorphic lens pair). This relay lens, as well as the position of all optical elements on the setup were carefully optimized using the software Zemax (Zemax, LLC) to maximize resolution and light throughput at the camera. This optimization led to slightly tilting the camera compared to the axis of the widefield image detection path. Angles between the fluorescence beams and the camera were 9° (direct path) and 16° (DMD path). Therefore the image guide was also slightly tilted (by an angle of 1°) compared to the optical axis.

#### Image guide and micro-objective

We used a 2.5-meter long image guide (FIGH-30-650S, Fujikura) composed of 30,000 individual step-index fibres with an intercore distance of 3.3 μm. Cores are distributed on a circle of diameter 600 μm, and the fibre is coated with a silicon resine to a total external diameter of 750 μm. In practice, the bundle was fixed in a home-made brass ferrule (1.25 mm diameter) with a fast UV curing optical adhesive (NOA81, Thorlabs), polished with fine grit sandpapers (12 μm to 1 μm) on a polisher from a shared neuroscience workshop facility (Neurofablab, Center for Psychiatry and Neuroscience, Paris), cleaned with isopropanol, and checked with a fibre microscope (FS201, Thorlabs).

A GRIN lens micro-objective was positioned near the exit surface of the image guide to perform imaging at a distant plane in the sample (working distance: 200 μm) and to improve lateral resolution (magnification of the objective: 2.6, intercore distance at the sample: 1.3 μm). Using this micro-objective, we imaged a field of view of 230 μm diameter with a lateral resolution (limited by Nyquist criteria) of 2.6 μm. The micro-objective was attached to the image guide using a custom-made connection device designed with SolidWorks (Dassault Systèmes) and 3D-printed in a biocompatible material (Titanium Alloy Ti6Al4V ELI, Strat Up Concept). This head mount was then attached to the skull with dental cement.

### *In vivo* experiments

#### Animal care

Experimental procedures were conducted in accordance with the institutional guidelines and in compliance with French and European laws and policies. All procedures were approved by the ‘Charles Darwin’ Ethics Committee (project number 04828.02).

#### Animal preparation

Six male mice C57BL/6JRj (Janvier Labs) of 8 weeks were used in this study. Each mouse was deeply anaesthetized by inhalation of isoflurane (3% for induction, 1.5–2% for maintenance) in 100% oxygen and then head-fixed in a stereotaxic frame. Meanwhile, a warming plate (37.5 to 38 °C) was used to maintain the mouse at physiological temperature. Eye ointment was applied to prevent from drying out. After intra-peritoneal injection of buprenorphine (0.015 mg/mL, 0.1mg per kg body weight, Buprecare) to reduce pain, we gently incised the skin and covered the skull with optiBond preparation (Kerr). A 3 mm diameter craniotomy was then performed over the cortex without damaging the dura. A thin glass coverslip (No 0, Warner Instruments) was positioned on the craniotomy and fixed to the skull with UV-cured dental cement (Tetric EvoFlow, Ivoclar Vivadent). 150–200 μL of a 5% w/v solution of rhodamine dextran (Rhodamine B Isothiocyanate-dextran 70000MW, Sigma-Aldrich) dissolved in saline was injected into the left retro-orbital sinus of the mouse using a 26 gauge needle. The head-mount connecting the image guide to the micro objective was then approached to the glass coverslip with a micromanipulator (PT3/M, Thorlabs) and attached to the skull with dental cement.

#### Imaging experiments

Acquisitions were first performed during anaesthetized conditions. Then, mice recovered from anaesthesia for 25min before acquisitions in awake conditions were performed. This relatively short recovery time was chosen in order to limit the total duration of the experiment, as dextran was continuously leaking out from the vessels and imaging contrast was slowly decreasing with time. During awake imaging sessions, no constraints were applied to the mice, and they could move freely in the cage. Their behaviour was recorded with a camera placed above the cage (acA1300-200uc, Basler).

### Data analysis

#### Computation of the differential multipoint-scanning confocal image

We first extracted from the raw image two ROIs corresponding to the multipoint-scanning confocal image and the widefield image. Theses images were then filtered with a uniform filter of size 3 pixels (1 μm) and the confocal image was stretched in x and y to the same dimensions than the widefield image. The lateral shift between the images was then found automatically using the DIPimage function findshift. When a stack of images was acquired (either a time lapse, a z-stack, or a “benchmark” series where the parameters of the pinhole patterns are changed), this operation was performed only once and the same shift was applied to the whole stack. In the case where the sample was a fluorescent plane, the multiplying factor applied to the widefield image before computation of the differential scanning confocal image could then be evaluated using equation 20. In the general case, the differential multipoint-scanning image could then be calculated using equation 11 (and after evaluation of this multiplication factor).

#### Computation of imaging contrast (blood vessel imaging)

A line was drawn orthogonal to the direction of the vessel of interest and the intensity profile was extracted. Images were low-pass filtered using a Gaussian filter of size *σ* = 1.5 pixels (0.5 μm) to improve signal to noise ratio. We defined *M* as the maximum intensity of the profile and *B* as the average value over the first and last 15% of the points of the profile, where no in-focus object was imaged. Therefore, *B* corresponded to the out-of-focus background defined in the theory section, while *M* was the sum of the out-of-focus background and the in-focus signal *S* (as defined in the theory section) coming from the microvessel of interest. The background to signal was then computed as *B*/(*M* − *B*).

## Electronic supplementary material

Movie 1

Supplementary Information

## References

[1] Yang W, Yuste R (2017). *In Vivo* Imaging of Neural Activity. Nature methods.

[2] Eberle AL, Selchow O, Thaler M, Zeidler D, Kirmse R (2015). Mission (im)possible - mapping the brain becomes a reality. Microsc..

[3] Carrillo-Reid, L., Yang, W., Kang Miller, J. E., Peterka, D. S. & Yuste, R. Imaging and Optically Manipulating Neuronal Ensembles. *Annu*. *review of biophysics* (2017). 10.1146/annurev-biophys-070816-033647 28301770

[4] Rost BR, Schneider-Warme F, Schmitz D, Hegemann P (2017). Optogenetic Tools for Subcellular Applications in Neuroscience. Neuron.

[5] Dombeck DA, Khabbaz AN, Collman F, Adelman TL, Tank DW (2007). Imaging Large-Scale Neural Activity with Cellular Resolution in Awake, Mobile Mice. Neuron.

[6] Ghosh KK (2011). Miniaturized Integration of a Fluorescence Microscope. Nat. Methods.

[7] Yu H, Senarathna J, Tyler BM, Thakor NV, Pathak AP (2015). Miniaturized optical neuroimaging in unrestrained animals. NeuroImage.

[8] Sawinski J., Wallace D. J., Greenberg D. S., Grossmann S., Denk W., Kerr J. N. D. (2009). Visually evoked activity in cortical cells imaged in freely moving animals. Proceedings of the National Academy of Sciences.

[9] Zong, W. *et al*. Fast High-Resolution Miniature Two-Photon Microscopy for Brain Imaging in Freely Behaving Mice. *Nature methods* **14**, 713–719 (2017). 10.1038/nmeth.4305 28553965

[10] Ferezou Isabelle, Bolea Sonia, Petersen Carl C.H. (2006). Visualizing the Cortical Representation of Whisker Touch: Voltage-Sensitive Dye Imaging in Freely Moving Mice. Neuron.

[11] Murayama M, Perez-Garci E, Luscher HR, Larkum ME (2007). Fiberoptic System for Recording Dendritic Calcium Signals in Layer 5 Neocortical Pyramidal Cells in Freely Moving Rats. J. of Neurophysiol..

[12] Flusberg Benjamin A, Nimmerjahn Axel, Cocker Eric D, Mukamel Eran A, Barretto Robert P J, Ko Tony H, Burns Laurie D, Jung Juergen C, Schnitzer Mark J (2008). High-speed, miniaturized fluorescence microscopy in freely moving mice. Nature Methods.

[13] Szabo V, Ventalon C, De Sars V, Bradley J, Emiliani V (2014). Spatially Selective Holographic Photoactivation and Functional Fluorescence Imaging in Freely Behaving Mice with a Fiberscope. Neuron.

[14] Lauritzen M (2001). Relationship of spikes, synaptic activity, and local changes of cerebral blood flow. *J*. *Cereb Blood Flow and Metab*.*: Off J*. *of the Int*. Soc. of Cereb. Blood Flow Metab..

[15] Rad MS (2017). Voltage and Calcium Imaging of Brain Activity. Biophys. J..

[16] Keller PJ, Ahrens MB (2015). Visualizing Whole-Brain Activity and Development at the Single-Cell Level Using Light-Sheet Microscopy. Neuron.

[17] Ziv Y, Ghosh KK (2015). Miniature microscopes for large-scale imaging of neuronal activity in freely behaving rodents. Curr. Opin. Neurobiol..

[18] Ziv Y (2013). Long-Term Dynamics of Ca1 Hippocampal Place Codes. Nat. Neurosci..

[19] Neil N, Wilson N (1998). & JUÅ kaitis, N. A light efficient optically sectioning microscope. J. Microsc..

[20] Heintzmann R, Hanley QS, Arndt-Jovin D, Jovin TM (2001). A dual path programmable array microscope (PAM): simultaneous acquisition of conjugate and non-conjugate images. J. Microsc..

[21] Mertz J (2011). Optical Sectioning Microscopy with Planar or Structured Illumination. Nat. methods.

[22] Petráň M, Hadravský M, Egger MD, Galambos R (1968). Tandem-Scanning Reflected-Light Microscope*. JOSA.

[23] Conchello JA, Lichtman JW (1994). Theoretical analysis of a rotating-disk partially confocal scanning microscope. Appl. Opt..

[24] Verveer N, Hanley N, Verbeek N, Vliet NV, Jovin N (1997). Theory of confocal fluorescence imaging in the programmable array microscope (PAM). J. Microsc..

[25] Mertz, J. *Introduction to Optical Microscopy* (W. H. Freeman, 2009).

[26] Martial Franck P., Hartell Nicholas A. (2012). Programmable Illumination and High-Speed, Multi-Wavelength, Confocal Microscopy Using a Digital Micromirror. PLoS ONE.

[27] Wilson T (2011). Resolution and optical sectioning in the confocal microscope. J. Microsc..

[28] Dubbs A, Guevara J, Yuste R (2016). moco: Fast Motion Correction for Calcium Imaging. Front. Neuroinformatics.

[29] Drew PJ, Blinder P, Cauwenberghs G, Shih AY, Kleinfeld D (2010). Rapid determination of particle velocity from space-time images using the Radon transform. J. Comput. Neurosci..

[30] Drew PJ, Shih AY, Kleinfeld D (2011). Fluctuating and sensory-induced vasodynamics in rodent cortex extend arteriole capacity. Proc. Natil. Acad. Sci. United States Am..

[31] Matz G, Messerschmidt B, Gross H (2016). Design and evaluation of new color-corrected rigid endomicroscopic high NA GRIN-objectives with a sub-micron resolution and large field of view. Opt. Express.

[32] Rosenblum WI (1969). Erythrocyte Velocity and a Velocity Pulse in Minute Blood Vessels on the Surface of the Mouse Brain. Cir. Res..

[33] Gmitro AF, Aziz D (1993). Confocal microscopy through a fiber-optic imaging bundle. Opt. Lett..

[34] Gobel W, Kerr JND, Nimmerjahn A, Helmchen F (2004). Miniaturized Two-Photon Microscope Based on a Flexible Coherent Fiber Bundle and a Gradient-Index Lens Objective. Opt. Lett..

[35] Santos, S. *et al*. Optically Sectioned Fluorescence Endomicroscopy with Hybrid-Illumination Imaging through a Flexible Fiber Bundle. *J*. *of Biomed*. *Opt*. **14** (2009). 10.1117/1.3130266 19566286

[36] Keahey P, Ramalingam P, Schmeler K, Richards-Kortum RR (2016). Differential structured illumination microendoscopy for *in vivo* imaging of molecular contrast agents. Proc. Natl. Acad. Sci..

[37] Sabharwal YS, Rouse AR, Donaldson L, Hopkins MF, Gmitro AF (1999). Slit-scanning confocal microendoscope for high-resolution *in vivo* imaging. Appl. Opt..

[38] Hughes M, Yang G-Z (2016). Line-scanning fiber bundle endomicroscopy with a virtual detector slit. Biomed. Opt. Express.

[39] Tang Y, Carns J, Richards-Kortum RR (2017). Line-scanning confocal microendoscope for nuclear morphometry imaging. J. Biomed. Opt..

[40] Risi MD, Makhlouf H, Rouse AR, Tanbakuchi AA, Gmitro AF (2014). Design and Performance of a Multi-Point Scan Confocal Microendoscope. Photonics.

[41] Bozinovic N, Ventalon C, Ford T, Mertz J (2008). Fluorescence Endomicroscopy with Structured Illumination. Opt. Express.

[42] Chen T-W (2013). Ultrasensitive Fluorescent Proteins for Imaging Neuronal Activity. Nat..

[43] Zhou, P. *et al*. Efficient and accurate extraction of *in vivo* calcium signals from microendoscopic video data. *eLife* **7**, 10.7554/eLife.28728 (2018). 10.7554/eLife.28728 PMC5871355 29469809

[44] Lu J (2018). MIN1pipe: A Miniscope 1-Photon-Based Calcium Imaging Signal Extraction Pipeline. Cell Reports.

[45] Dipoppa M (2018). Vision and Locomotion Shape the Interactions between Neuron Types in Mouse Visual Cortex. Neuron.

[46] Chen X (2012). Diffraction of digital micromirror device gratings and its effect on properties of tunable fiber lasers. Appl. Opt..

