# Supplementary material for: Fast confocal fluorescence imaging in freely behaving mice
Source: Sci Rep. 2018 Nov 2;8:16262. doi: 10.1038/s41598-018-34472-x (PMC6214968; doi:10.1038/s41598-018-34472-x)
Supplement: Supplementary file 2 — Supplementary Information [file 41598_2018_34472_MOESM2_ESM.pdf]

Fast confocal fluorescence imaging in freely behaving mice: Supplementary Information

Clara Dussaux[1], Vivien Szabo[2,+], Yan Chastagnier[2,+], Jozsua Fodor[1,+], Jean-François Léger[1], Laurent Bourdieu[1], Julie Perroy[2], and Cathie Ventalon[1,*]

[1]Institut de biologie de l'École normale supérieure (IBENS), École Normale Supérieure, CNRS, INSERM, PSL Research University, 46 rue d'Ulm, Paris, 75005, France
[2]Institut de Génomique Fonctionnelle (IGF), CNRS, INSERM, University of Montpellier, 141 rue de la Cardonille, Montpellier, 34094, France
[*]cathie.ventalon@ens.fr
[+]these authors contributed equally to this work

ABSTRACT

This supplementary information section contains supplementary text, methods, figures, and movie

Supplementary Text

Analysis and reduction of the speckle noise

We characterized the 3D intensity distribution of the illumination patterns (Fig. 1A, left). These beams show strong spatial inhomogeneities due to the coherent nature of our light source and transmission through the image guide consisting of multimode individual fibers. These spatial inhomogeneities can produce acquisition noise if objects are moving or if the speckle pattern is changing with time. If out-of-focus objects are moving, the resulting intensity variation will be reduced by the detection PSF. Therefore, the most unfavorable cases (leading to the largest amount of noise) are obtained when in-focus objects are moving, or when the speckle pattern is changing with time, both situations that should produce the same level of noise. In particular, variation of the speckle pattern with time occurs when the fiber bundle is displaced, *ie.* when the mouse moves. To quantify this noise, we placed a fluorescent plane at the focal plane of the GRIN-lens objective and performed time-lapse differential multipoint scanning confocal imaging while agitating vigorously the fiber bundle, mimicking the movements of the mouse. The measured noise was about 7% with the acquisition parameters used in the manuscript for vasculature imaging (5ms exposure time, $A = 7.5\mu$m and $P = 5$), and ranged between 6% and 9% depending on the parameters used ($A = [4.5\mu\text{m}; 7.5\mu\text{m}; 12\mu\text{m}]$; $P = [3; 4; 5; 7; 10]$; exposure time: $5 - 18$ms)(Fig.1B, top). These results were reproducible among several similar acquisitions. This level of noise was not a limiting factor in our experiments and we could easily detect the highly contrasted red blood cells and measure their velocities.

For experiments requiring lower acquisition noise, devices that rapidly change the speckle pattern with time can be used. The speckle noise is then reduced by averaging out multiple speckle patterns. With our optical setup, during acquisition of an image, each point in the imaged plane is only illuminated by one pinhole pattern (or in practice, 2 or 3 to reduce grid artifacts) for a time that can be as short as 62μs (corresponding to the maximum frame rate of the DMD). Therefore, speckle patterns should be modified faster than this short timescale. We tested one device composed of a deformable mirror and a multimode fiber (Albedo System, high frequency version, Errol Laser) that allows projecting successive intensity patterns with a resulting speckle contrast of 3% on a 62μs time window (Fig.1C). Transmission of the device is about 50%. We introduced this system on our optical setup and illuminated the DMD with these fast varying speckle patterns. We then measured the 3D intensity distribution of the illumination patterns and found that the inhomogeneities were strongly reduced (Fig.1A, right). Finally, we measured the noise obtained when imaging an in-focus fluorescent plane while agitating vigorously the fiber bundle and found that this noise was reduced to about 3% with the parameters used from imaging microvasculature (5ms exposure time, $A = 7.5\mu$m and $P = 5$). Noise ranged between 2% and 4% depending on the parameters used ($A = [4.5\mu\text{m}; 7.5\mu\text{m}; 12\mu\text{m}]$; $P = [3; 4; 5; 7; 10]$; exposure time: $5 - 18$ms)(Fig.1B, bottom), corresponding to an improvement of a factor 2.5 compared with our regular setup. We expect further reduction of the remaining noise if a multimode fiber of larger diameter or larger NA was used in the Albedo System, resulting in smaller speckle grains at the image guide.

Maximum imaging rate

The maximum imaging speed can be limited either by the refreshing rate of the DMD ($f_{\text{DMD}} = 16.4$kHz) or by the acquisition speed of the camera. During acquisition of a single image on the camera, the DMD must display at least P^2 images to achieve illumination of the whole field of view. However, in practice we chose to display $2P^2$ or $3P^2$ images (by shifting each set of P^2 patterns by $1/2$ or $1/3$ of an individual pinhole size along the diagonal) to reduce pattern artifacts. Therefore, the maximum acquisition speed allowed by the DMD is equal to $f_{\text{DMD}}/(2P^2) = f_{\text{DMD}}D/2$ (when displaying $2P^2$ images). For $P = 5$ *ie.* $D = 4\%$, we obtain a maximum acquisition speed of 330Hz. Regarding the limit imposed by the camera, because readout is provided using two rolling shutters covering half of the camera chip, the camera maximum acquisition rate is $2/(T_{\text{lcam}}H)$ (where H is the number of image lines and $T_{\text{lcam}} = 10\mu$s is the read time for one line). We designed the optical setup such that the diameter of the image guide (containing roughly 300 individual fibers) spans approximately $H = 600$ camera lines. Therefore, the maximum acquisition rate of the camera is 330Hz. By putting the two constraints together, we conclude that for $D > 4\%$, the maximum acquisition rate is limited by the camera to 330Hz, whereas for $D < 4\%$ it is limited by the DMD to $D * 8200$Hz.

Influence of the size of detection pinholes

We investigated the influence of the detection pinhole size on the performances of differential multipoint-scanning confocal imaging (Fig. 3). We found that increasing the size of the detection pinholes while keeping the illumination pinholes at a fixed size allows increasing the in-focus signal with a limited increase of the section thickness, compared to the situation where both pinholes have the same size (Fig. 3A-B). However, this comes with an increase in the background for regular multipoint-scanning confocal imaging (Fig. 3C) and therefore an increase of the noise for differential multipoint-scanning confocal imaging, and was not used in practice.

Calculation of the signal to noise ratio for differential multipoint-scanning confocal imaging

In this section, we calculated the signal to noise ratio as a function of the pinhole density D under the following hypotheses: (1) We considered that $S_{\text{conf}} \approx \gamma S_{\text{wide}}$. γ is the attenuation factor measured with the fluorescent plane but we can assume that the same factor applies for any in-focus sample. (2) Because in our experiments the maximum illumination power at the sample is not limited by photobleaching or photodamage but rather by the total available illumination power (especially for small values of D), we considered a fixed incident laser power at the DMD and a variable illumination intensity at the sample $I_i(D) = DI_{i,D=1}$. Under these assumptions, the widefield signal can be expressed as : $S_{\text{wide}}(D) = DS_{\text{wide,D=1}}$, where $S_{\text{wide,D=1}}$ represents the signal coming from the focal plane that would be detected with widefield imaging for $D = 1$. Using equation (12) of the main text, we can then write the differential multipoint-scanning confocal signal as:

$$S_{\text{diff}} = \alpha I_{d,\text{diff}} \tag{1}$$

$$= \alpha T_{\text{conf}}(\gamma - D) D S_{\text{wide,D=1}} \tag{2}$$

and the noise can be derived from equation (14). We finally obtain the expression of the signal to noise ratio for differential multipoint-scanning confocal imaging:

$$(S/N)_{\text{diff}} = f(D)\sqrt{\alpha S_{\text{wide,D=1}}} \tag{3}$$

where we defined $f(D)$ as:

$$f(D) = \frac{\sqrt{T_{\text{conf}}}(\gamma - D)\sqrt{D}}{\sqrt{\gamma + D^2 T_{\text{conf}}/T_{\text{wide}} + D(1 + DT_{\text{conf}}/T_{\text{wide}})B/S_{\text{wide}}}} \tag{4}$$

We plotted $f(D)$ on Figure 4 in the case of microvasculature imaging ($B/S_{\text{wide}} = 4.6$, as measured in our experiments), for a pinhole size of $A = 7.5\mu$m (corresponding to $\gamma = 0.2$), and for $T_{\text{conf}}/T_{\text{wide}} = 4.5$. $f(D)$ shows a maximum for $D = 0.04$, which is therefore the value taken for all experiments in the paper.

We can also compare the signal and the noise in regular and differential multipoint-scanning confocal imaging, using the same parameters. Using equation (12) of the main text and considering once again that $S_{\text{conf}} \approx \gamma S_{\text{wide}}$, we obtain that the relative loss of signal with differential *versus* regular multipoint-scanning confocal imaging is equal to $D/\gamma = 0.04/0.2 = 0.2$. The ratio between the standard deviations of the noise obtained in differential and regular multipoint-scanning confocal imaging can be calculated as:

$$\frac{\sigma_{d,\text{diff}}}{\sigma_{d,\text{conf}}} = \sqrt{\frac{\gamma + D^2 T_{\text{conf}}/T_{\text{wide}} + D(1 + DT_{\text{conf}}/T_{\text{wide}})B/S_{\text{wide}}}{\gamma + DB/S_{\text{wide}}}} \tag{5}$$

For the parameters used here, we find $\sigma_{d,\text{diff}}/\sigma_{d,\text{conf}} = 1.05$.

Supplementary Methods

Simulations

Simulations were performed using Matlab R2013A (Mathworks) and DIPimage toolbox (http://www.diplib.org/). The PSF was calculated using the Stokseth approximation[1]. Simulation parameters: NA $= 0.5$, $A = 7.5\mu$m, $D = 1/4^2$. Aberrations were not considered in the calculation.

Optical setup

Design and optimization

The DMD can be modeled as a 2 dimensional blazed grating, with a diffraction efficiency into the main order strongly dependent on the incident angle and the wavelength[2]. Therefore, we chose to separate the laser and fluorescence beams on the surface of the DMD and optimize their angles independently (see main Fig. 2). For the laser beam, the angle between the beam reflected at the DMD and the normal to the DMD is equal to 13.5°. For the fluorescence beam, the angle between the beam incident on the DMD and the normal to the DMD is equal to 9°. These angles allow reaching diffraction efficiency into the main order close to 60% for both beams, which is the maximum value that can be achieved with this DMD. Using this configuration, illumination and detection pinholes are located on two sides of the DMD and are independent from one another. Therefore, the size of detection pinholes can be made slightly larger than that of illumination pinholes to increase collection efficiency. In addition, an incident angle of 13.5° is also suitable for a 488nm laser beam. Therefore a second laser beam colinear with the 561nm laser beam could be used, for example, for photoactivation of Channelrhodopsin 2.

A 561nm laser beam (LMX-561L-500-COL-PP, Oxxius) is expanded using a telescope composed of two achromatic doublets (of focal lengths 25mm and 200mm, AC127-025-A-ML and AC254-200-A-ML, Thorlabs) and illuminates the left part of the DMD. The DMD is rotated by 45° around its normal such that the reflected beam (in the main diffraction order) lies in the horizontal plane. The DMD is imaged onto the entrance surface of the image guide using a tube lens ($f_1 = 150$mm, DLB-20-150PM, Optosigma), and a 10X microscope objective (UPLSAPO 10X2, Olympus). In this way, illumination grid patterns displayed at the DMD are projected onto the entrance surface of the image guide, transported in the image guide and then reimaged in the sample using a micro-objective (01 NEM-100-25-10-860-DS-ST, Grintech). Fluorescence collected by the micro-objective is imaged onto the image guide and transported to the microscope, where it is split into two parts using a 90/10 beamsplitter (21011, 90/10 Beamsplitter - UF2, Chroma) placed after the objective. 90% of the fluorescence beam is imaged (using the objective and a tube lens ($f_2 = 150$mm, DLB-20-150PM, Optosigma)) onto the right part of the DMD, displaying a pattern of detection pinholes. Fluorescence light reflected at the DMD is then imaged on the left part of the camera using a relay lens composed of 2 identical relay lenses ($f_3 = f_4 = 150$mm, AC508-150-A, Thorlabs), forming a multipoint-scanning confocal image. The remaining 10% of fluorescence light transmitted by the 90/10 beamsplitter is directly imaged onto the right part of the camera (using the microscope objective and a tube lens ($f_5 = 125$mm, AC254-125-A-ML, Thorlabs)) forming a conventional widefield image. Confocal and widefield images are acquired simultaneously. Emissions filters are placed on the two detection paths to reject residual laser light.

The detection beam path between the DMD and the camera was carefully optimized, by taking into account one important constraint, namely that detection efficiency of the camera drops for incident angles larger than 18° (data not shown). This is due to the structure of sCMOS pixels, that contain a microlens to focus light on a small detector. For large incidence angle, a part of light does not reach the sensitive area. Therefore, we made sure that the incidence angle on the camera for the multipoint-scanning confocal image was smaller than 18°. Since the angle between the DMD surface and the fluorescence beam reflected at the DMD is larger than this value (33°), we used the off-axis relay lens formed by lenses L_3 and L_4 to partly straighten up the image (anamorphic lens pair). This relay lens, as well as the position of all optical elements on the setup were carefully optimized using the software Zemax (Zemax, LLC) to maximize resolution and light throughput at the camera. This optimization led to slightly tilting the camera compared to the axis of the widefield image detection path. Angles between the fluorescence beams and the camera were 9° (direct path) and 16° (DMD path). Therefore the image guide was also slightly tilted (by an angle of 1°) compared to the optical axis.

Alignment protocol

Careful optical alignment of the setup was performed in order to simultaneously observe two well-contrasted images of the image guide on the camera (*i.e.* individual fibers of the image guide were seen with high contrast). Then, we used the software for data acquisition to align the detection pinholes with the illumination pinholes. The x- and y-offsets and the x- and y-stretches of the detection pinhole pattern were optimized by using an in-focus fluorescent plane as the sample, a fixed illumination pinhole pattern and by maximizing light throughput at the camera.

Image guide and micro-objective

We used a 2.5-meter long image guide (FIGH-30-650S, Fujikura) composed of 30,000 individual step-index fibers with an intercore distance of 3.3μm. Cores are distributed on a circle of diameter 600μm, and the fiber is coated with a silicon resine

to a total external diameter of 750μm. In practice, the bundle was fixed in a home-made brass ferrule (1.25mm diameter) with a fast UV curing optical adhesive (NOA81, Thorlabs), polished with fine grit sandpapers (12μm to 1μm) on a polisher from a shared neuroscience workshop facility (Neurofablab, Center for Psychiatry and Neuroscience, Paris), cleaned with isopropanol, and checked with a fiber microscope (FS201, Thorlabs).

A GRIN lens micro-objective was positioned near the exit surface of the image guide to perform imaging at a distant plane in the sample (working distance: 200μm) and to improve lateral resolution (magnification of the objective: 2.6, intercore distance at the sample: 1.3μm). Using this micro-objective, we imaged a field of view of 230μm diameter with a lateral resolution (limited by Nyquist criteria) of 2.6μm. The micro-objective was attached to the image guide using a custom-made connection device designed with SolidWorks (Dassault Systèmes) and 3D-printed in a biocompatible material (Titanium Alloy Ti6Al4V ELI, Strat Up Concept). This head mount was then attached to the skull with dental cement.

Characterization of the optical sectioning and measurement of the point-spread-function
To characterize the optical sectioning, we used fluorescent planes made of glass coverslips spin-coated with a 20μL droplet of a solution of 1mM of Rhodamine B (R6626-25G, Sigma, $> 95\%$) and 10% w/v Poly Methy Methacrylate (PMMA) (445746-25G, Sigma) dissolved in chloroform. Rotation of the spin coater was 6100rpm for 30s. The fluorescent plane was placed below the micro-objective and attached to a piezoelectric motor (PILine M 663.465, PI). Axial stacks were registered with a step of 2μm.

To characterize the fiberscope PSF, we used the same protocol as described by Zong et al[3]. 2μL of Tetaspeck 1 micron Microspheres (T7282, ThermoFisher Scientific) were first diluted into 100μL phosphate-buffered saline and then mixed with 900μL low melting agarose (1.5%). A droplet of the agarose solution containing the microsphere fluorescent beads was applied between two coverglasses. The sample was placed below the micro-objective and attached to a piezoelectric motor (PILine M 663.465, PI). Axial stacks were registered with a step of 1μm.

To measure illumination power at the sample, a power meter (PM100 USB, Thorlabs) was placed just below the micro-objective such that all illumination light was collected.

Characterization of the 3D intensity distribution of the illumination patterns
Illumination patterns were characterized with a custom-made transmission microscope. A thin layer of rhodamine spin-coated on a microscope coverslip (see above) was placed at the vicinity of the imaging plane of the micro-objective and imaged onto a CMOS camera (acA1920-40um, Basler) using a high-NA microscope objective (40X, NA=0.75, Zeiss) and a tube lens (f = 200 mm, achromatic doublet, AC254-200-A-ML, Thorlabs). The fiberscope probe (micro-objective and image guide) was mounted on a piezoelectric motor (PILine M 663.465, PI) to allow shifts in the distance between the probe and the fluorescent layer. 200 z-sections of the illumination beams were then obtained, with axial steps of 2μm.

Optical setup for illumination with a fast varying speckle pattern
The 561nm laser beam is expanded using a telescope composed of two lenses (of focal lengths 50 mm and 100 mm) and illuminates the deformable mirror of the Albedo system (high frequency version, Errol laser). The exit surface of the square shaped multimode fiber (NA = 0.22, size $= 175\mu$m) is then imaged onto the DMD using a telescope composed of two lenses (of focal lengths 8 mm and 250 mm). The remaining part of the fiberscope system is left unchanged. The illumination beam at the DMD is characterized by introducing a mirror right before the DMD and positioning a camera at the image of the exit surface of the multimode fiber.

In vivo experiments
Animal care
Experimental procedures were conducted in conformity with the institutional guidelines and in compliance with French and European laws and policies. All procedures were approved by the ‘Charles Darwin’ Ethics Committee (project number 04828.02).

Animal preparation
Six male mice C57BL/6JRj (Janvier Labs) of 8 weeks were used in this study. Each mouse was deeply anesthetized by inhalation of isoflurane (3% for induction, $1.5-2\%$ for maintenance) in 100% oxygen and then head-fixed in a stereotaxic frame. Meanwhile, a warming plate (37.5 to 38°C) was used to maintain the mouse at physiological temperature. Eye ointment was applied to prevent from drying out. After intra-peritoneal injection of buprenorphine (0.015mg/mL, 0.1mg per kg body weight, Buprecare) to reduce pain, we gently incised the skin and covered the skull with optiBond preparation (Kerr). A 3mm diameter craniotomy was then performed over the cortex without damaging the dura. A thin glass coverslip (No 0, Warner Instruments) was positioned on the craniotomy and fixed to the skull with UV-cured dental cement (Tetric EvoFlow, Ivoclar Vivadent). $150-200\mu$L of a 5% w/v solution of rhodamine dextran (Rhodamine B Isothiocyanate-dextran 70000MW, Sigma-Aldrich) dissolved in saline was injected into the left retro-orbital sinus of the mouse using a 26 gauge

needle. The head-mount connecting the image guide to the micro objective was then approached to the glass coverslip with a micromanipulator (PT3/M, Thorlabs) and attached to the skull with dental cement.

Imaging experiments

Acquisitions were first performed during anesthetized conditions. Then, mice recovered from anesthesia for 25min before acquisitions in awake conditions were performed. During awake imaging sessions, no constraints were applied to the mice, and they could move freely in the cage. Their behavior was recorded with a camera placed above the cage (acA1300-200uc, Basler).

Data analysis

Image analysis was performed off-line using Matlab R2013A (Mathworks) and DIPimage toolbox.

Computation of the differential multipoint-scanning confocal image

We first extracted from the raw image two ROIs corresponding to the multipoint-scanning confocal image and the widefield image. Theses images were then filtered with a uniform filter of size 3 pixels (1μm) and the confocal image was stretched in x and y to the same dimensions than the widefield image. The lateral shift between the images was then found automatically using the DIPimage function findshift. When a stack of images was acquired (either a time lapse, a z-stack, or a ”benchmark” series where the parameters of the pinhole patterns are changed), this operation was performed only once and the same shift was applied to the whole stack. In the case where the sample was a fluorescent plane, the multiplying factor applied to the widefield image before computation of the differential scanning confocal image could then be evaluated using equation 20 of the main text. In the general case, the differential multipoint-scanning image could then be calculated using equation 11 of the main text (and after evaluation of this multiplication factor).

Computation of imaging contrast (blood vessel imaging)

A line was drawn orthogonal to the direction of the vessel of interest and the intensity profile was extracted. Images were low-pass filtered using a gaussian filter of size $\sigma = 1.5$ pixels (0.5μm) to improve signal to noise ratio. We defined M as the maximum intensity of the profile and B as the average value over the first and last 15% of the points of the profile, where no in-focus object was imaged. Therefore, B corresponded to the out-of-focus background defined in the theory section, while M was the sum of the out-of-focus background and the in-focus signal S (as defined in the theory section) coming from the microvessel of interest. The background to signal was then computed as $B/(M-B)$.

Motion correction

The ImageJ plugin MOCO[4] was used to correct motion artifacts. To improve processing speed, images were downsampled by a factor of two, converted to 8 bit and cropped. A gaussian filter (size $\sigma = 2$ pixels) was also applied twice in order to eliminate the footprint of the fiber cores. The matrix of lateral drifts across time was then extracted by running MOCO and used to correct motion on the original movie.

Measurement of RBC speed in individual vessels

Individual vessels were selected for analysis by manually drawing curved lines following the shape of the vessels on the average image of a time series (with a thickness adjusted to the vessel diameter). x-t profiles corresponding to selected individual vessels were then extracted from the original time-series. On these profiles, bright and dark streaks correspond to labeled plasma and non-fluorescent RBCs, respectively. The angle θ_S of these streaks (compared to the vertical axis) is directly related to the RBC velocity v[5]:

$$v = \frac{\Delta x}{\Delta t}\frac{1}{\tan(\theta_S)} \tag{6}$$

where Δx is the pixel size (0.36μm) and Δt is the time between 2 frames. To improve the precision of velocity measurement, the x-t profiles were filtered with a Sobel filter of size 3 or 5, to obtain a signal $F(x,t)$[6]. The radon transform $R(r,\theta)$ of this signal was then computed using the radon function from Matlab[5]:

$$R(r,\theta) = \frac{1}{LT}\int_0^L \int_0^T F(x,t)\delta\left(r - \frac{x}{\Delta x}\cos(\theta) - \frac{\mathrm{t}}{\Delta \mathrm{t}}\cos(\theta)\right) dt dx \tag{7}$$

For each angle θ we then compute the variance $V(\theta)$ along the radius r, and θ_S corresponds to the angle maximizing $V(\theta)$.

Because our camera was used in the rolling shutter mode, images lines were not acquired exactly at the same time: acquisition started at the central line and then moved up and down simultaneously at a speed of 9.7μs per line. We took this effect into account by calculating a corrected value of the velocity: $v_{\mathrm{corr}} = l/(v/l + \Delta T_{\mathrm{rs}})$, where v is determined using equation 6, l is the length of the ROI and ΔT_{rs} is the time delay between acquisition of the two ends of the ROI due to the rolling shutter.

Strictly speaking, this correction assumes that the ROI is a straight line, which is reasonable in practice - especially considering that the correction factor is small (difference between v and v_{corr} is only a few percents).

Finally, we used two joint criteria to assess data validity. First, the velocity should be smaller than the maximum velocity that we are able to measure taking into account the frame rate f and ROI length l: $v_{max} = lf/2$ (see main text). Second, following data analysis from[5], we calculated the ratio between the maximum variance and the mean variance. We empirically found that a ratio > 5 was necessary for accurate detection.

References

1. Stokseth, P. A. Properties of a Defocused Optical System. *J. Opt. Soc. Am.* **59**, 1314–1321 (1969). DOI 10.1364/JOSA.59.001314.
2. Chen, X. *et al.* Diffraction of digital micromirror device gratings and its effect on properties of tunable fiber lasers. *Appl. Opt.* **51**, 7214–7220 (2012). DOI 10.1364/AO.51.007214.
3. Zong, W. *et al.* Fast High-Resolution Miniature Two-Photon Microscopy for Brain Imaging in Freely Behaving Mice. *Nat. methods* **14**, 713–719 (2017).
4. Dubbs, A., Guevara, J. & Yuste, R. moco: Fast Motion Correction for Calcium Imaging. *Front. Neuroinformatics* **10**, 6 (2016). DOI 10.3389/fninf.2016.00006.
5. Drew, P. J., Blinder, P., Cauwenberghs, G., Shih, A. Y. & Kleinfeld, D. Rapid determination of particle velocity from space-time images using the Radon transform. *J. Comput. Neurosci.* **29**, 5–11 (2010). DOI 10.1007/s10827-009-0159-1.
6. Chhatbar, P. Y. & Kara, P. Improved blood velocity measurements with a hybrid image filtering and iterative Radon transform algorithm. *Front. Neurosci.* **7**, 106 (2013). DOI 10.3389/fnins.2013.00106.

Supplementary Movie

Movie 1. Differential multipoint-scanning confocal imaging of microvasculature in the cortex of a freely behaving mouse. Exposure time: 5ms. $A = 6\mu$m. $D = 0.04$. Movie play-back speed is 40Hz.

Supplementary Figures

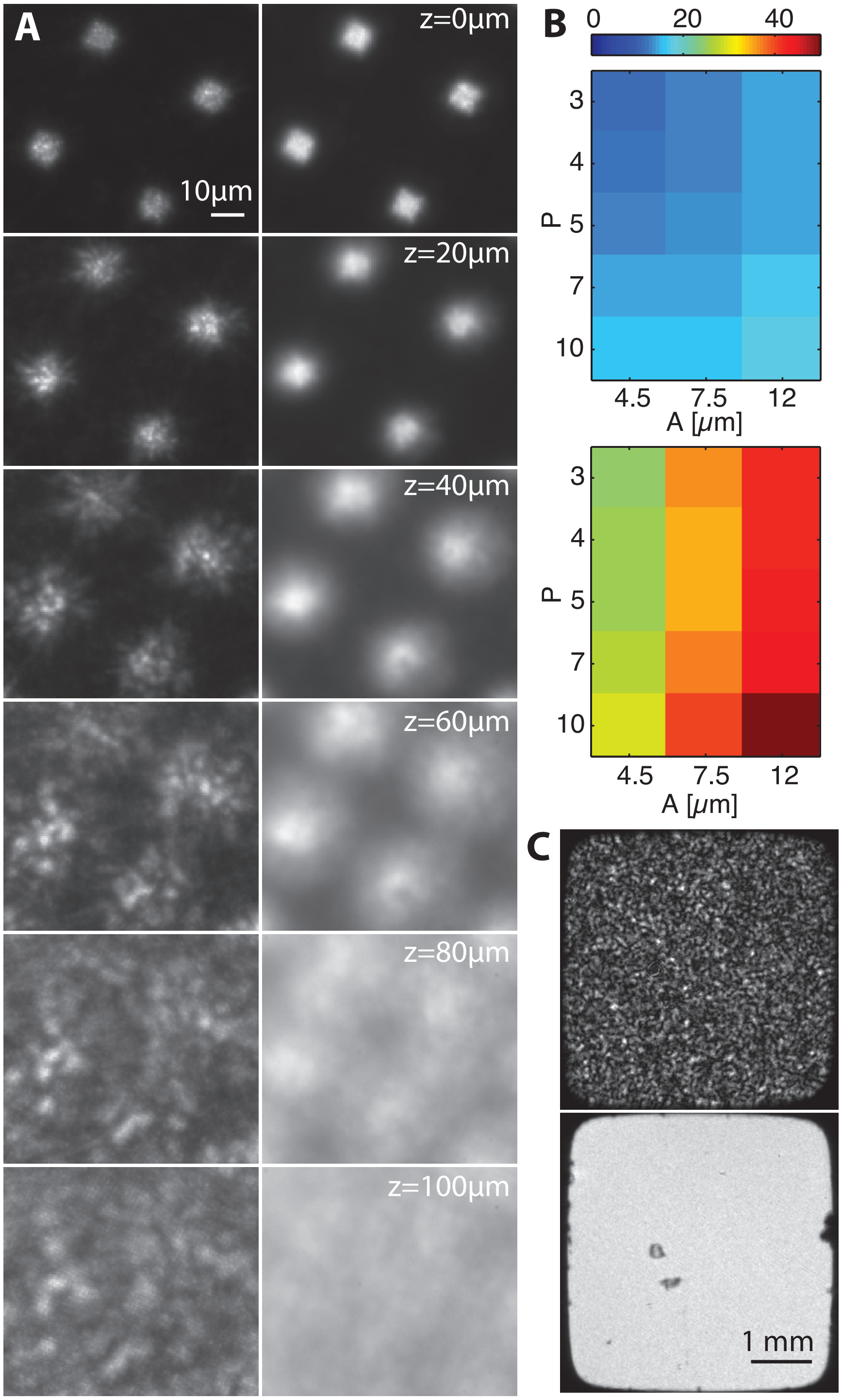

Figure 1. A. Sections of the illumination patterns along the x-y planes at different axial positions z (indicated on the images). These sections are measured with a transmission microscope described in SI Methods. Left: patterns obtained with the optical setup described in the main text. Right: patterns obtained when rapidly modifying the speckle pattern with the albedo system. Parameters: $A = 7.5\mu$m. $P = 5$. B. Signal to noise ratio measured with differential multipoint scanning confocal time-lapse imaging when rapidly moving the image guide, for a set of parameters A and P. Exposure time was 5ms for $P = [3;4;5]$, 9ms for $P = 7$ and 19ms for $P = 10$. In the last two cases, this corresponded to the shortest possible exposure time, limited by the DMD frame rate. Top: optical setup as described in main text. Bottom: the albedo system is introduced to rapidly modify the speckle pattern. C. Characterization of the illumination beam at the DMD using the albedo system with the system off (top) and on (bottom).

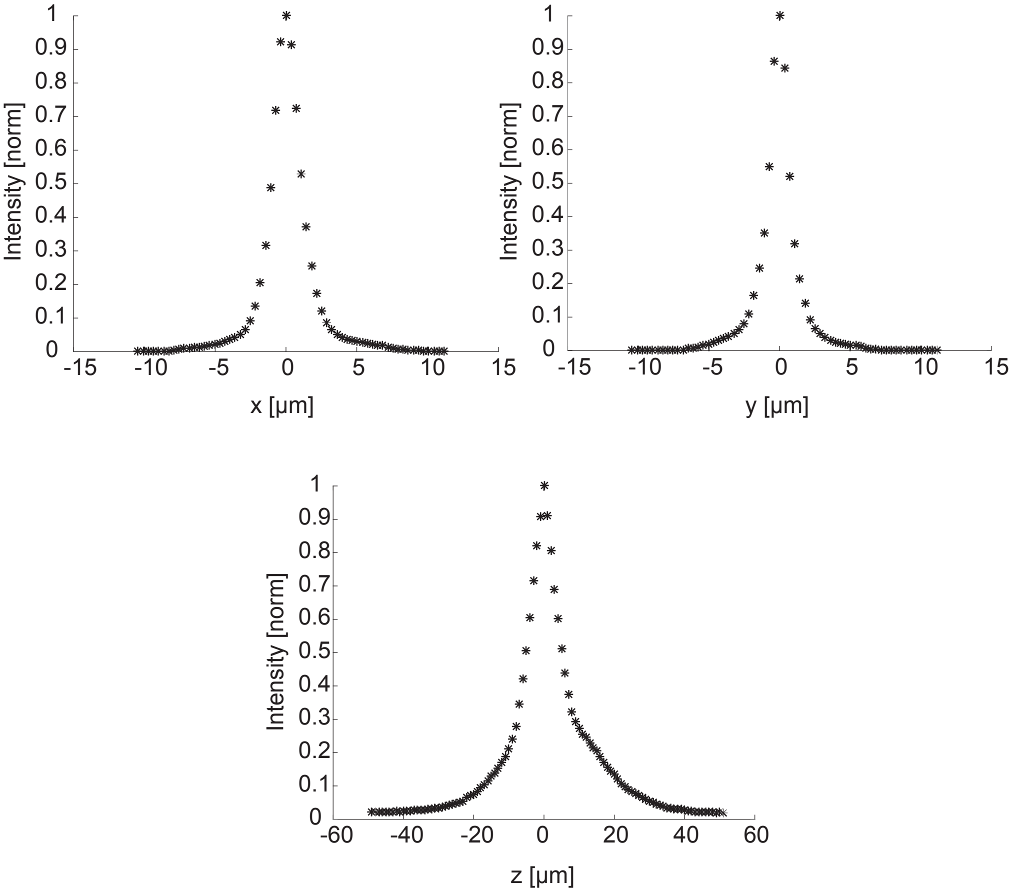

Figure 2. Point-spread function of the differential multipoint scanning confocal fiberscope. Profiles along x (top left), y (top right) and z (bottom) averaged over 50 measurements performed with 50 different beads located at different positions on the field of view. The corresponding resolutions (FWHM) are: $2.2 \pm 0.2\mu$m along x; $1.6 \pm 0.3\mu$m along y; $10.5 \pm 2.1\mu$m along z. Parameters: $A = 7.5\mu$m, $P = 5$.

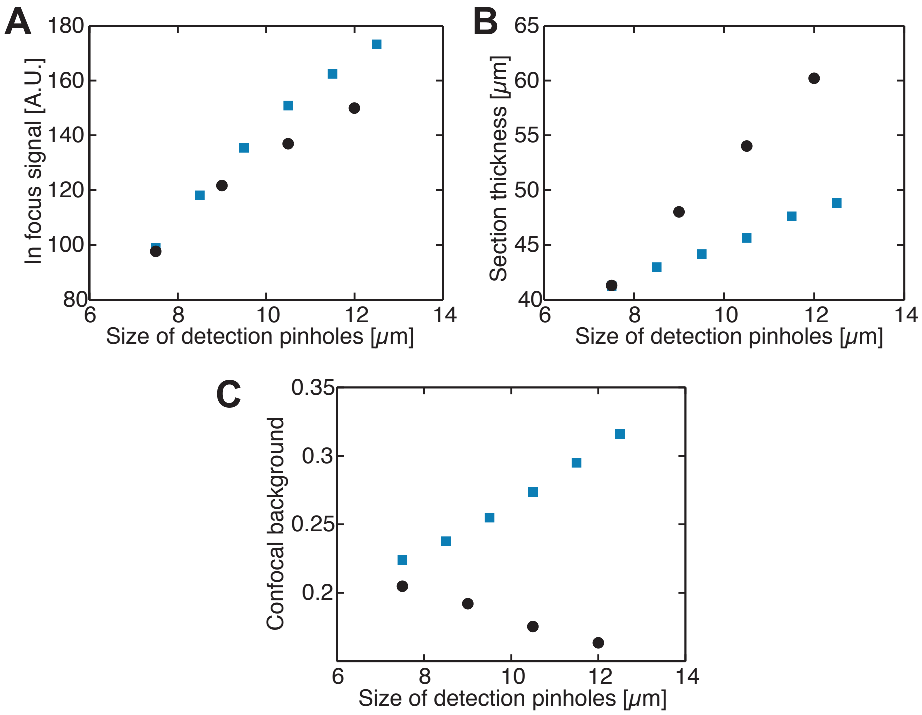

Figure 3. Characterization of the imaging performances as a function of the size of detection pinholes, in two different situations: when illumination and detection pinholes have identical sizes (black circles) and when detection pinholes are larger than illumination pinholes (blue squares). In this latter case, illumination pinholes have a fixed size of 7.5μm. A. Differential multipoint-scanning confocal signal obtained with a fluorescent plane in-focus. B. Thickness of the optical section obtained with differential multipoint scanning confocal imaging. C. Background for regular multipoint-scanning confocal imaging. To measure this background, we plotted the ratio between the sectioning curves measured with regular multipoint-scanning confocal imaging and widefield imaging (normalized to 1 at $z = 0$, when the plane is in-focus) similar to dotted blue line in Fig 3B (main text).The background is the offset of this curve.

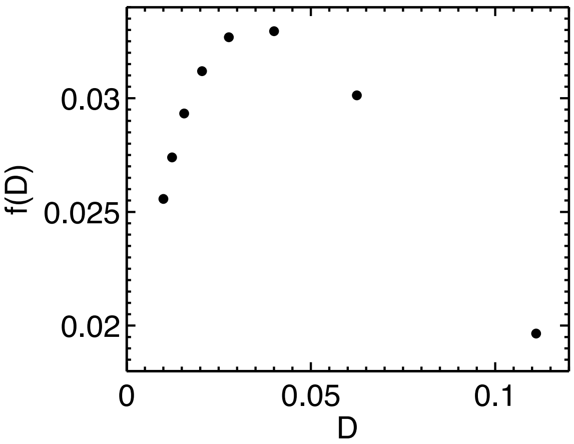

Figure 4. Signal to noise ratio for differential multipoint-scanning confocal imaging as a function of the pinhole density. The value of $f(D)$ (equations 3 and 4) is calculated using the parameters of the current setup: $T_{\text{conf}} = 0.45$, $T_{\text{wide}} = 0.1$, and $\gamma = 0.2$ (corresponding to $A = 7.5$). We used the background to signal ratio measured in our experiments when performing micro-vasculature imaging ($B/S_{\text{wide}} = 4.6$). A maximum value is obtained for $D = 0.04$.

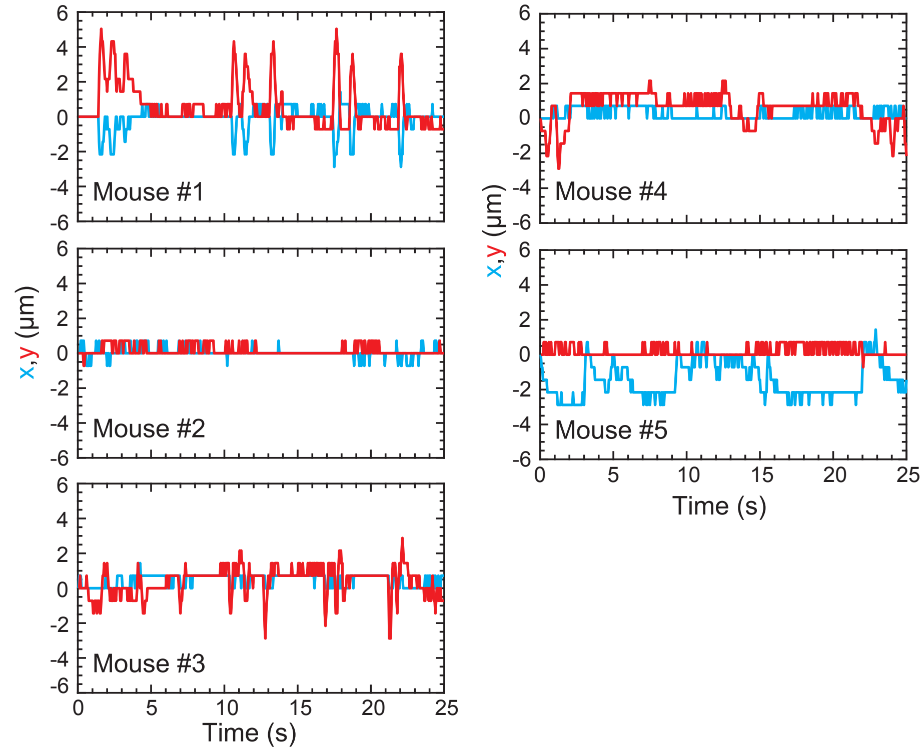

Figure 5. Lateral movements of the field-of-view along x (blue lines) and y (red lines) during free behavior measured in 5 mice using differential multipoint-scanning confocal imaging. Acquisition rate: 20Hz. $A = 7.5\mu$m. $D = 0.04$.
